# Heat Shock Protein 90: From Molecular Chaperone Function to Therapeutic Targeting in Malignancies

**DOI:** 10.1002/advs.75895

**Published:** 2026-06-03

**Authors:** Beibei Zhang, Xinxin Zou, Jiansong Zhou, Qinmiao Sun, Dahua Chen

**Affiliations:** ^1^ Institute of Biomedical Research Yunnan University Kunming Yunnan China; ^2^ Southwest United Graduate School Kunming China; ^3^ State Key Laboratory of Organ Regeneration and Reconstruction Institute of Zoology Chinese Academy of Sciences Beijing China; ^4^ Beijing Institute for Stem Cell and Regenerative Medicine Beijing China; ^5^ School of Life Sciences University of Chinese Academy of Sciences Beijing China

**Keywords:** cancer recurrence, drug resistance, heat shock protein, immunotherapy, molecular chaperone

## Abstract

Heat shock protein 90 (HSP90) is an evolutionarily conserved molecular chaperone that occupies a central position in cellular proteostasis and adaptation to stress. By promoting protein folding and facilitating the functional activation of a broad repertoire of clients, HSP90 underpins essential cellular processes, including development, proliferation, differentiation, and stress responses. In cancer, this extensive network renders tumor cells highly dependent on HSP90 to maintain oncogenic signaling, tolerate proteotoxic stress, and survive therapeutic insults. In this review, we propose an integrated conceptual framework linking HSP90's molecular chaperone functions to its pathological roles in cancer: HSP90 serves as a central node that concurrently supports oncogenic signaling, buffers proteotoxic stress, maintains cancer stem cell plasticity, and shapes tumor‐immune interactions‐all of which converge to drive therapeutic resistance and tumor recurrence. Within this framework, we summarize the molecular mechanisms governing HSP90 activity and dissect its context‐dependent roles in cancer progression, drug resistance, and immune regulation. We further highlight recent advances in HSP90‐targeted interventions, including small‐molecule inhibitors, monoclonal antibodies, engineered immune cells, and emerging strategies designed to prevent, delay, or overcome drug resistance. Taken together, these developments underscore HSP90 as a versatile therapeutic node and point toward innovative, resistance‐aware strategies for clinical translation and future research.

## Graphical abstract

In this review, an integrated conceptual framework linking HSP90's molecular chaperone functions to its pathological roles in cancer is proposed. HSP90 serves as a central node that integrates oncogenic signaling, buffers proteotoxic stress, maintains cancer stem cell plasticity, and shapes tumor‐immune interactions, all of which converge to drive therapeutic resistance and tumor recurrence. Within this framework, recent advances are further highlighted in HSP90‐targeted interventions, including small‐molecule inhibitors, monoclonal antibodies, engineered immune cells, and emerging strategies specifically designed to prevent, delay, or overcome drug resistance and cancer recurrence.

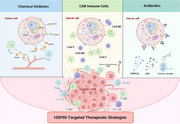

## 1 Introduction

Heat shock proteins (HSPs) are essential molecular chaperones that preserve protein homeostasis under both physiological and stress conditions [1, 2, 3]. In disease states, particularly within the tumor microenvironment (TME), which is characterized by hypoxia, nutrient deprivation, acidosis, chronic inflammation, and exposure to chemotherapeutic agents, proteotoxic stress is heightened, and HSPs are robustly induced to buffer damage and sustain cell viability [1, 2, 3, 4]. In this context, HSPs do not merely act as passive protectors of proteome integrity but become active enablers of malignant progression and therapeutic failure. By stabilizing mutated, overexpressed, or misfolded oncoproteins and stress‐survival factors, they help tumor cells withstand cytotoxic chemotherapy, targeted agents, endocrine therapies, radiotherapy, and immunotherapies, thereby driving both primary refractoriness and acquired drug resistance [5, 6].

Among them, the 90 kDa heat shock protein (HSP90) has emerged as a central node in the chaperone network and a compelling therapeutic target [3, 7]. HSP90 stabilizes and activates more than 300 client proteins, including key oncogenic kinases, transcription factors, DNA damage response mediators, and steroid hormone receptors that collectively sustain proliferative signaling, confer resistance to apoptosis, and promote invasion and metastasis [8, 9]. Many of these clients are canonical resistance determinants‐for example, kinase drivers and downstream effectors that reactivate signaling when upstream nodes are inhibited, or DNA repair factors that blunt the cytotoxicity of genotoxic drugs [10, 11, 12]. In addition, HSP90 supports the function and stability of drug efflux transporters and anti‐apoptotic proteins, thereby fostering multidrug‐resistant phenotypes [12]. Through these clients, HSP90 integrates multiple cancer hallmarks and underpins a form of “non‐oncogene addiction” in which tumor cells rely on stress‐support pathways to tolerate both oncogenic and therapy‐induced stress and to maintain a drug‐resistant state [13, 14].

Despite this strong biological rationale, early clinical development of HSP90 inhibitors, most notably geldanamycin (GA) and its derivatives, was hampered by dose‐limiting systemic toxicities and the unintended activation of a compensatory heat shock response (HSR) [15]. Pharmacologic inhibition of HSP90 disrupts its repressive interaction with client proteins and further activates heat shock factor 1 (HSF1) to drive the transcription of additional chaperones. This HSR is fundamentally cytoprotective: it enhances global chaperone capacity, stabilizes alternative survival pathways, diminishes the antitumor efficacy of HSP90 blockade, and can foster adaptive drug resistance [16]. Tumor cells can thus escape HSP90 inhibition by shifting their chaperone dependency toward HSP70/HSP27, rewiring signaling networks, activating autophagy or proteasome pathways to clear damaged proteins, or enriching for cancer stem‐like cells that intrinsically resist cytotoxic stress [17, 18, 19]. Together, these mechanisms have limited the depth and durability of responses to HSP90 inhibitors and highlight drug resistance as a central obstacle in HSP90‐directed therapy.

In response, contemporary strategies have broadened beyond classical N‐terminal (NT) ATP‐competitive inhibitors. These include the development of C‐terminal (CT) HSP90 inhibitors and allosteric modulators that are less prone to triggering a robust HSR; derivatives of natural products with improved pharmacological properties; and isoform‐ or compartment‐selective agents that preferentially target specific HSP90 paralogs (e.g., HSP90α/β, GRP94, TRAP1) [1, 20]. Such refinements aim not only to reduce systemic toxicity but also to attenuate HSF1‐driven compensatory responses and thereby delay or prevent resistance. Parallel efforts focus on improving therapeutic index through targeted delivery platforms‐such as nanoparticles, liposomes, and ligand‐ or antibody‐directed conjugates‐that concentrate HSP90 inhibitors in tumor tissue while reducing systemic exposure and efflux‐mediated escape [21]. In addition, rational combination strategies seek to co‐target HSP90 and critical downstream or parallel pathways, including oncogenic signaling cascades, DNA damage responses, and immune checkpoints, often in conjunction with chemotherapy, targeted therapies, radiotherapy, or immunotherapies [22, 23, 24]. By simultaneously disrupting HSP90 clients that mediate survival and resistance, and by blocking compensatory escape routes (e.g., co‐inhibition of HSF1, HSP70, autophagy, or key pro‐survival kinases), these combinatorial approaches aim to exploit the broad client spectrum of HSP90, prevent network rewiring, and delay or overcome both primary and acquired drug resistance.

This review summarizes recent advances in HSP90‐targeted therapies, emphasizing their mechanistic basis and therapeutic applications across cancer and other diseases. We compare how differences in proteomic dependencies‐particularly HSP90's contributions to oncogenesis, drug resistance, and immune regulation‐intersect with distinct TME features across malignancies to shape the efficacy and limitations of HSP90‐directed interventions. While previous reviews have laid a solid foundation for​ HSP90‐targeted therapy and the development of associated inhibitors [7, 8, 9], our work specifically aims to bridge the gap between fundamental mechanistic insights and translational research, with a particular focus on overcoming drug resistance. With several HSP90 inhibitors advancing through (pre‐) clinical trials, we provide a timely analysis of the current landscape and critically evaluate the evolution of these inhibitors, discuss strategies to overcome early limitations like toxicity, and explore their potential to reverse resistance to conventional and targeted therapies, including the related delivery system and the chaperone mis‐translocation‐based immunotherapy. We particularly discuss how these context‐specific dependencies and resistance mechanisms can inform the design of optimal therapeutic regimens, including the selection of effective combination partners, and their impact on the emergence of both primary and acquired resistance. Finally, we discuss future directions for integrating HSP90 modulation into multimodal precision strategies aimed at preventing or reversing drug‐resistant disease.

## 2 The Biological Roles of HSP90 and Their Structural Basis for Proteostasis

HSPs are grouped into six major families based on their approximate molecular mass and structural characteristics: HSP100, HSP90, HSP70, HSP60, HSP40, and the small HSPs (sHSPs, e.g., HSP27/HSP20) [25]. HSP90 is one of the most abundant molecular chaperones in eukaryotic cells, constituting ∼1%–3% of the total cellular protein content under basal conditions [26, 27, 28, 29, 30, 31]. Its expression is further elevated in many tumor types, where HSP90 is detected not only in the cytosol but also at the plasma membrane and within the extracellular milieu [32]. The HSP90 family comprises several isoforms with distinct subcellular localizations and partially specialized functions [33]: the cytosolic isoforms HSP90α and HSP90β, the endoplasmic reticulum (ER)‐resident glucose‐regulated protein 94 (GRP94), and the mitochondrial paralog tumor necrosis factor receptor‐associated protein 1 (TRAP1). These isoforms form a compartmentalized chaperone network that maintains proteostasis across key organelles. These diverse biological functions are intimately linked to the unique structural architecture and ATP‐dependent mechanistic cycle of HSP90.

**FIGURE 1:**
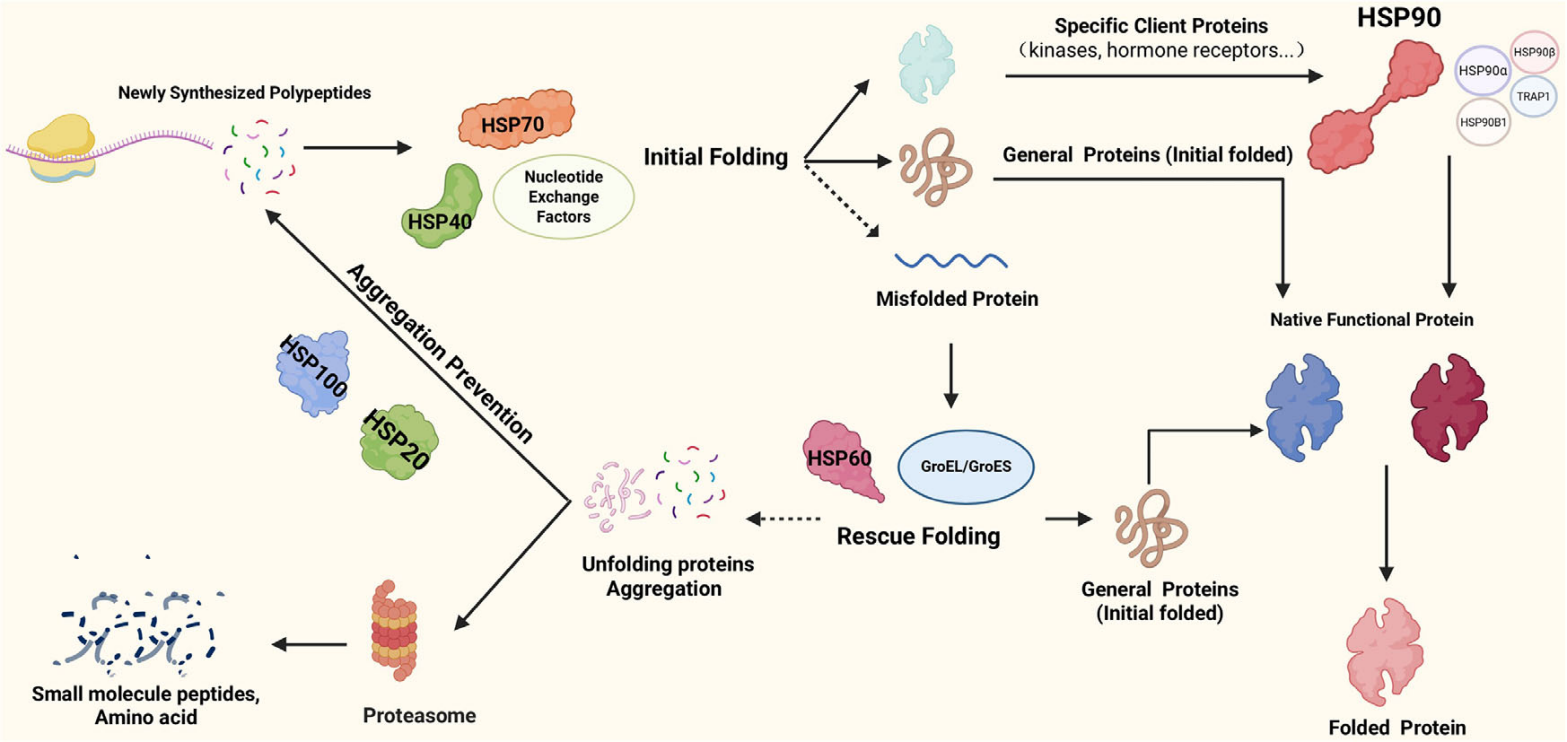
The cooperative mechanisms of HSPs mediated proteostasis.

Under physiological conditions, HSP90 proteins operate as central components of the proteostasis network that spans the cytosol, endoplasmic reticulum, and mitochondria. For example, GRP94 (GP96/HSP90B1), an ER‐resident HSP90 paralog, along with co‐chaperones including BiP (GRP78/HSPA5), facilitates the folding and maturation of secretory and membrane proteins and contributes to the recognition of misfolded proteins by ER stress (ERS) sensors [34]. As summarized in Figure 1, the different HSP families, including HSP90 cooperate in a coordinated cascade to preserve proteome integrity. Nascent chains are first engaged by the HSP70 system (together with HSP40) [35]. They can then be transferred either to the HSP60 chaperone system for productive folding or to HSP90 for specialized maturation of selected clients [36]. HSP90 operates downstream or in parallel to these early folding steps and is specialized for the maturation of selected client proteins, including many signaling molecules. By interacting with a network of co‐chaperones and using ATP, HSP90 stabilizes client proteins in near‐native conformations, thereby ensuring their proper activity, subcellular localization, and turnover. In addition, cells deploy a surveillance and repair machinery for damaged or aggregated proteins. Small HSPs (e.g., HSP27) act as a first line of defense by binding partially unfolded proteins and preventing aggregation [37], while the HSP100 system can disaggregate preformed aggregates [38]. Proteins that remain irreversibly misfolded are typically ubiquitinated and degraded by the proteasome or routed to autophagy‐lysosomal pathways, thereby recycling amino acids and preventing proteotoxic stress.

**FIGURE 2:**
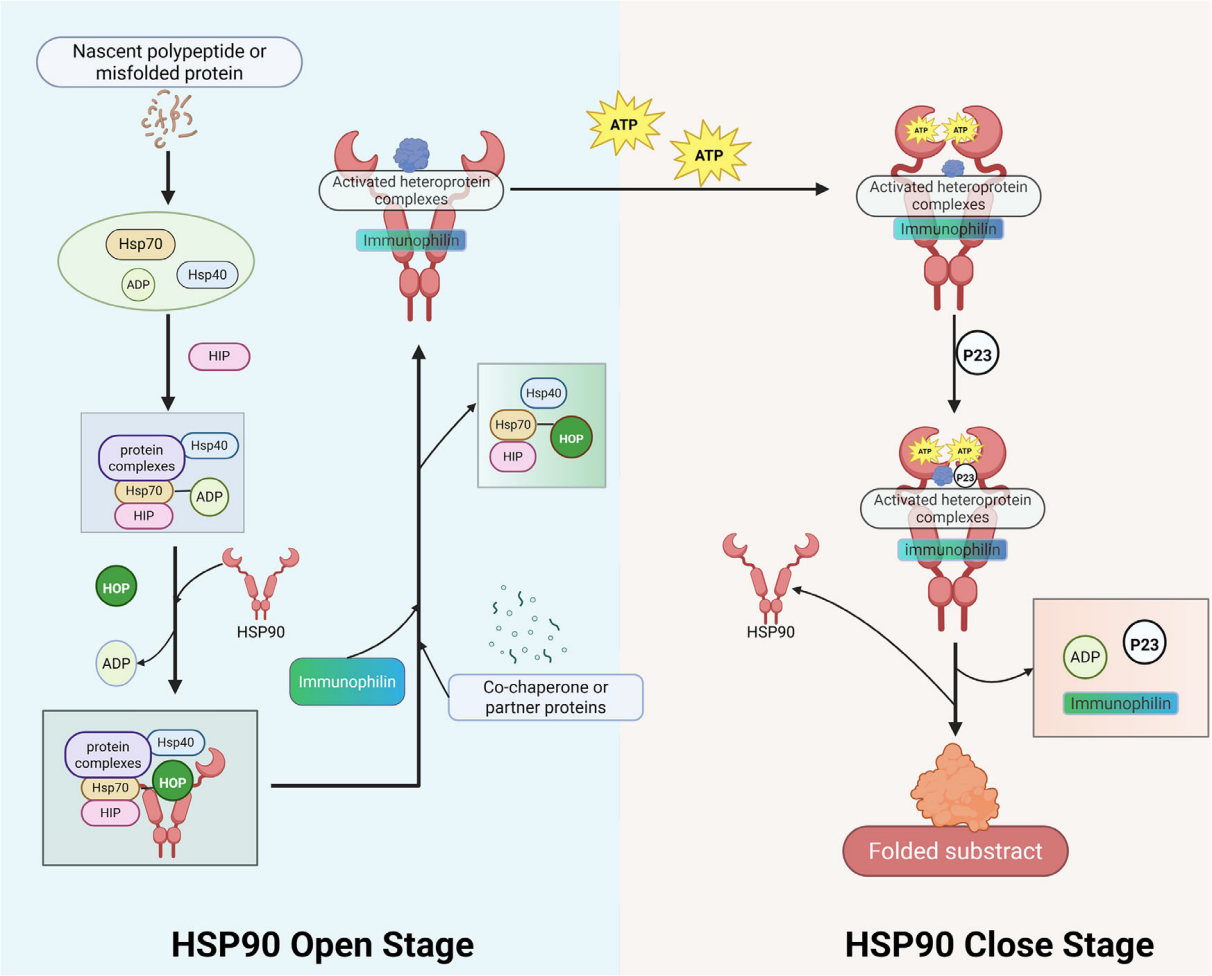
HSP90 couples intrinsic ATPase with co‐chaperones to remodel client protein conformations.

It is evident that the HSP90 family plays a unique and crucial biological role in protein homeostasis, which is closely linked to its protein structure. Structurally, HSP90 functions as an obligate homodimer. Each monomer comprises three conserved domains arranged linearly: an N‐terminal domain (NTD), a middle domain (MD), and a C‐terminal domain (CTD), connected by a flexible, charged linker [39]. The NTD contains the ATP‐binding pocket, which is essential for driving the ATPase cycle that underlies HSP90's conformational dynamics. ATP binding and hydrolysis at the NTD power a series of coordinated domain movements that switch the chaperone between “open” and “closed” conformations. The MD is primarily responsible for client engagement and participates in ATP hydrolysis by contributing catalytic residues. The CTD mediates constitutive dimerization and harbors the conserved MEEVD motif, which serves as a docking site for tetratricopeptide repeat (TPR)‐containing co‐chaperones [39]. These structural elements together create a versatile platform for client recognition, folding, and quality control. As illustrated in Figure 2, the HSP90 functional cycle is tightly coupled to ATP binding and hydrolysis and is regulated by an extensive co‐chaperone network. In its nucleotide‐free or ADP‐bound state, HSP90 adopts a “relaxed” open conformation. ATP binding to the NTD induces N‐terminal dimerization and a transition to a more “compact” closed state, which clamps onto client proteins [40]. This step is the primary target of classical NTD‐directed HSP90 inhibitors, which compete with ATP, lock the chaperone in an inactive conformation, and ultimately promote proteasomal degradation of client proteins [41]. Client loading and maturation require the coordinated action of multiple co‐chaperones. HSP70 and its co‐chaperones initially engage nascent or partially unfolded clients and then hand them off to HSP90 via the HSP70/HSP90 organizing protein (HOP), which simultaneously binds HSP70 and the CTD of HSP90. Additional co‐chaperones, such as CDC37, preferentially recruit protein kinases; immunophilins regulate hormone receptor complexes; and P23 binds to the ATP‐bound closed state of HSP90, stabilizing this conformation and promoting final client maturation. Aha1 interacts with the MD to stimulate ATP hydrolysis, accelerating the chaperone cycle and facilitating client release. Once ATP is hydrolyzed, HSP90 reverts to an open conformation, and the folded client dissociates or is routed to downstream quality control pathways if folding fails.

HSPs, including HSP90, function as crucial molecular chaperones that safeguard protein homeostasis by ensuring correct protein conformation and stability, thereby protecting cells against proteotoxic stresses under both normal and adverse conditions [42, 43]. When cells experience severe or irreversible stress, chaperones can be actively released into the extracellular microenvironment or exposed to the cell surface along with the cell death process, acting as damage‐associated molecular patterns (DAMPs) [44]. In this response, heat shock transcription factors (primarily HSF1) sense proteotoxic stress and trigger a broad transcriptional reprogramming that increases the expression of heat shock genes. In the resting state, HSF1 exists as an inactive monomer bound to molecular chaperones such as HSP90 [45]. When ER stress occurs, proteasome function is impaired or misfolded proteins accumulate excessively, these chaperones are competitively recruited to the surface of aberrant proteins, leading to the dissociation and release of HSF1. Subsequently, released HSF1 rapidly undergoes trimerization, nuclear translocation, hyperphosphorylation, and deubiquitination modifications, forming a complex with high transcriptional activity [46, 47]. By binding to heat shock elements, in the promoter regions of target genes, this complex initiates the transcriptional reprogramming of molecular chaperones, proteasome subunits, and autophagy‑related genes, thereby coordinately restoring cellular protein homeostasis [48, 49]. The resulting accumulation of HSPs enhances the folding capacity of the cell, buffers protein damage, and promotes survival under adverse conditions [50].

In summary, the modular domain architecture of HSP90, its ATP‐driven conformational cycle, and its extensive co‐chaperone network collectively enable precise regulation of client protein maturation, stability, and activity. This structural and mechanistic versatility underpins HSP90's essential role as a master regulator of proteostasis and explains why its inhibition can simultaneously impact multiple oncogenic pathways, making HSP90 a particularly attractive yet challenging target for therapeutic intervention.

## 3 HSP90 Pathological Roles in Malignant Disease

With the structural framework and basic biological roles established, this section details the context‐dependent roles of HSP90 across major malignancy types, focusing on how it stabilizes oncogenic clients and drives disease progression. HSP90 acts as a central pathological mediator in multiple diseases by sustaining the integrity and activity of misfolded or aberrant client proteins, thereby disrupting cellular proteostasis as well as amplifying maladaptive stress responses. Its pathological impact is particularly pronounced and well‐characterized in cancer. Cancer cells typically experience heightened ERS due to extensive genetic mutations, copy number alterations, oxidative damage, hypoxia, and nutrient deprivation. Consequently, HSP90 is commonly upregulated in malignancies, supporting neoplastic proliferation, invasion, and metastasis through the stabilization of a broad spectrum of oncogenic client proteins. In cancer, multiple malignant cells co‐opt the HSP90 chaperone machinery to maintain the stability of numerous oncoproteins, a phenomenon consistent with the concept of “oncogene addiction”.

### 3.1 Breast Cancer

Breast cancer (BC) is a highly heterogeneous disease marked by aberrant HSP90 activity and overexpression, which contribute to tumor initiation, progression, and therapy resistance [51]. The diverse molecular subtypes of BC exhibit distinct dependencies on HSP90 for cell survival as well as proliferation, with its role being particularly pronounced in triple‐negative BC (TNBC) as well as HER2‐positive BC [52]. HSP90 plays an especially critical role in TNBC, as these tumors often rely on alternative oncogenic pathways that are heavily regulated by HSP90 [53]. HSP90 plays a crucial stabilizing role for non‐hormone‐dependent oncogenic signaling proteins, such as EGFR, MET, and STAT3, all of which are its client proteins. Consequently, HSP90 is increasingly considered a promising focus for therapeutic intervention in TNBC. For instance, the novel CT inhibitor SL‐145 suppresses metastatic TNBC without inducing an HSR by inhibiting oncogenic pathways, including AKT, JAK2/STAT3, and MEK/ERK, with degradation of clients like AKT, thereby reducing tumor growth, angiogenesis, and metastasis [54]. Another CT inhibitor, NCT‐547, targets cancer stem‐like subpopulations in TNBC by disrupting AKT, MEK, and STAT3 signaling, decreasing ALDH1 expression and CD44+/CD24− phenotypes, and inhibiting tumor growth without causing acute toxicity [55]. Additional compounds, such as the cyclic peptide AD05, also exhibit potent anti‐TNBC activity through HSP90 inhibition and induction of tumor cell death [56]. Furthermore, extracts from Calotropis procera latex have been shown to induce apoptosis and necrosis in TNBC cells without triggering an HSR, highlighting their potential as HSP90‐targeting agents [57]. Moreover, targeting the HSP90‐HIF1α pathway using SNX2112‐encapsulated nano‐micelles has shown promise in augmenting the efficacy of photothermal‐photodynamic therapy for TNBC [58]. Whereas in hormone receptor‐positive BC, HSP90 chaperones the estrogen receptor (ER) itself and its client proteins (HER2, AKT). Targeting HSP90 can promote ER degradation, offering a strategy to overcome endocrine resistance [59]. For example, silmitasertib (CX‐4945) interferes with the ERα/HSP90 interaction as well as promotes ERα proteolysis via CK2β inhibition, effectively reducing ERα protein levels and inhibiting proliferation in both tamoxifen‐susceptible as well as‐resistant cells [59]. Novel HSP90 inhibitors with senolytic activity, such as K4 and K5, have been identified in hormone‐induced senescence models, effectively reducing senescent cell (SnC) populations and increasing cell death [60]. Additionally, dual ER and HSP90 inhibitors, such as compound 755435, are being explored as promising multitarget agents to combat resistance in ER+ BC [61]. Moreover, in HER2‐positive BC, HSP90 serves as a critical chaperone for the HER2 receptor tyrosine kinase (RTK), a key oncogenic driver [62]. Resistance to trastuzumab is a significant clinical problem in HER2‐positive BC. The CT HSP90 inhibitor NCT‐547 induced apoptosis in both trastuzumab‐sensitive as well as trastuzumab‐resistant HER2‐positive BC cells by degrading full‐length and truncated HER2, reducing HER2 family heterodimerization, and impairing cancer stem‐like properties [63]. A Phase I trial reported that combining ganetespib with paclitaxel and trastuzumab achieved an overall 22% response rate and 56% stable disease in patients with trastuzumab‐refractory HER2‐positive metastatic BC [64]. Another trial using the HSP90 inhibitor retaspimycin HCl (IPI‐504) with trastuzumab resulted in stable disease in 62% of patients, although no confirmed responses were observed, potentially due to underdosing [65]. Mechanisms of resistance to ganetespib in HER2‐positive BC appear to involve augmented HSP expression alongside phospho‐activation of RTKs, particularly involving AKT stabilization, suggesting that co‐targeting AKT could enhance therapeutic efficacy [66]. Particularly, by stabilizing HER2 and preventing its degradation, HSP90 maintains signaling activity, thereby promoting cancer cell proliferation as well as survival [67]. Notably, HSP90 expression levels are upregulated in HER2‐positive models, suggesting a compensatory mechanism in which cancer cells enhance HSP90 activity to sustain oncogenic signaling pathways [62].

### 3.2 Lung Cancer

Lung cancer appears particularly responsive to HSP90 inhibition [68], a strategy that has shown augmented therapeutic effect when used with doxorubicin in both in vitro as well as in vivo models [69]. Lung cancer's dependence on HSP90 is highlighted by driver mutations like EGFR, which generate unstable oncoproteins requiring HSP90 for both stability and function [70]. Pharmacological targeting of HSP90 using agents like 17‐AAG disrupts key pathways, including the HIF‐1α/HSP90 interaction, thereby suppressing HIF‐1α accumulation even in radioresistant cells [71]. Furthermore, HSP90 stabilizes AKT, modulating the AKT/mTOR signaling axis to influence autophagy and support tumor cell survival [72].

### 3.3 Gastrointestinal Cancer

HSP90 is crucial in various gastrointestinal cancers, stabilizing hypoxia factors and metabolic enzymes. In colorectal cancer (CRC), it regulates survival and resistance, with high Hsp90α/β levels linked to poor prognosis, especially with BRAF‐V600E or HER2/neu [73]. A study reveals icaritin (ICA) induces CRC cell apoptosis by disrupting HSP90‐TXNDC9 interactions, suppressing autophagy. Targeted delivery of ICA with low‐temperature photothermal therapy (PTT) enhances anti‐tumor action [74]. In gastric cancer (GC) and hepatocellular carcinoma (HCC), HSP90 often collaborates with specific co‐chaperones or engages noncanonical pathways to support malignancy [75]. Moreover, the HSP90 inhibitor IPI‐493 demonstrated substantial efficacy in human gastrointestinal stromal tumors (GIST) xenograft models harboring diverse KIT mutations, leading to tumor stabilization, induction of apoptosis, and downregulation of KIT expression. It synergized with TKIs such as imatinib and sunitinib, enhancing anti‐tumor responses, particularly with sunitinib [76]. HSP90 facilitates metastasis and cancer stem cell (CSC) properties in GC by altering glycolytic enzyme localization through cytoskeletal interactions, thereby boosting glycolysis and EMT [77].

### 3.4 Ovarian Cancer

In ovarian cancer (OC), elevated HSP90 expression is linked to advanced disease stages and poorer patient outcomes, suggesting its utility as an independent prognostic indicator [78]. The expression of mixed lineage kinase 4β in OC cells is modulated by HSP90 inhibition as well as by heat and osmotic stress, highlighting its involvement in cellular stress response pathways [79]. Preclinical studies further demonstrate that the HSP90 inhibitor ganetespib acts synergistically with carboplatin, enhancing selective cytotoxicity against OC cells [80]. Ganetespib disrupts HSP90 activity, impairing DNA repair protein expression and cell cycle regulation, thereby enhancing radiation‐induced DNA damage and increasing talazoparib sensitivity in high‐grade serous OC cells [81]. Collectively, these findings suggest that targeting HSP90 may expand the therapeutic applicability of PARP inhibitors, even in homologous recombination‐proficient ovarian malignancies.

### 3.5 Prostate Cancer

Prostate cancer (PCa) progression, particularly toward the castration‐resistant stage, is often sustained by persistent androgen receptor (AR) signaling, which relies on HSP90‐mediated stabilization [82]. HSP90 stabilizes various kinases beyond AR, as evidenced by reduced PKD2, PKD3, and AKT levels following its pharmacological inhibition. Disrupting HSP90‐PKD3 binding specifically induces PKD3 degradation, establishing it as a crucial HSP90 client protein in metastasis progression [83]. HSP90 also plays roles in the extracellular milieu of PCa, and plasma levels of extracellular HSP90α (eHSP90α) are markedly higher in patients than in controls, with more than a two‐fold increase observed in metastatic cases and elevated levels in high T‐stage lesions, supporting its potential as both a biomarker as well as therapeutic target for PCa progression [84].

### 3.6 Hematological Malignancy

HSP90 is essential for the survival of hematological cancer cells, stabilizing key oncoproteins and transcription factors that drive proliferation and resistance to apoptosis, in multiple myeloma (MM) [85]. Targeting the HSF1‐HSP90 axis with triptolide or minnelide induces apoptosis, reduces HSP90‐dependent kinases, and inhibits chronic lymphocytic leukemia (CLL) progression, demonstrating therapeutic promise for hematologic malignancies [86]. Research in acute myeloid leukemia (AML) is investigating targeted HSP90 inhibitor delivery approaches. A6 peptide‐functionalized biomimetic nano‐particles loaded with HSP90 inhibitor G2111 demonstrate enhanced targeting efficacy and potent anti‐leukemic activity in AML models by exploiting overexpressed HSP90 and CD44 on malignant cells [87]. Overall, HSP90, along with other HSPs, serves as a versatile therapeutic target. The HSP90 inhibitor KW‐2478 depleted BCR‐ABL protein levels and overcame imatinib resistance associated with BCR‐ABL amplification. It synergized with imatinib, exhibiting stronger effects on imatinib‐resistant chronic myeloid leukemia (CML) cells and prolonging survival in mouse models, indicating a promising strategy for TKI‐resistant CML [88].

### 3.7 Other Nonmalignant Diseases

Beyond its well‐established role in oncology, HSP90 is critically involved in the progression of a wide range of nonmalignant diseases, highlighting its fundamental importance in cellular proteostasis and stress response. For example, in neurodegenerative disorders, including Parkinson's, Alzheimer's, as well as Huntington's diseases, HSP90 and its co‐chaperones are key regulators of protein folding, helping to prevent the aggregation of misfolded proteins that drive disease pathology [89]. Dysregulation of these chaperones can exacerbate disease progression [89]. For instance, HSP90 can prevent aggregation of zinc‐free P53, a protein implicated in various neurodegenerative pathologies [90]. Furthermore, inhibition of HSP90 with agents such as 17‐AAG has been shown to attenuate Aβ‐induced synaptic toxicity as well as memory deficits in Alzheimer's disease models, likely through activation of synaptic protein expression mediated by HSF1‐dependent transcriptional pathways [91]. Recent findings indicate that all‐trans retinoic acid upregulates cell surface HSP90 on human microglia, mediating tau protein internalization and degradation‐a potential strategy for Alzheimer's disease and related tauopathies [92].

**FIGURE 3:**
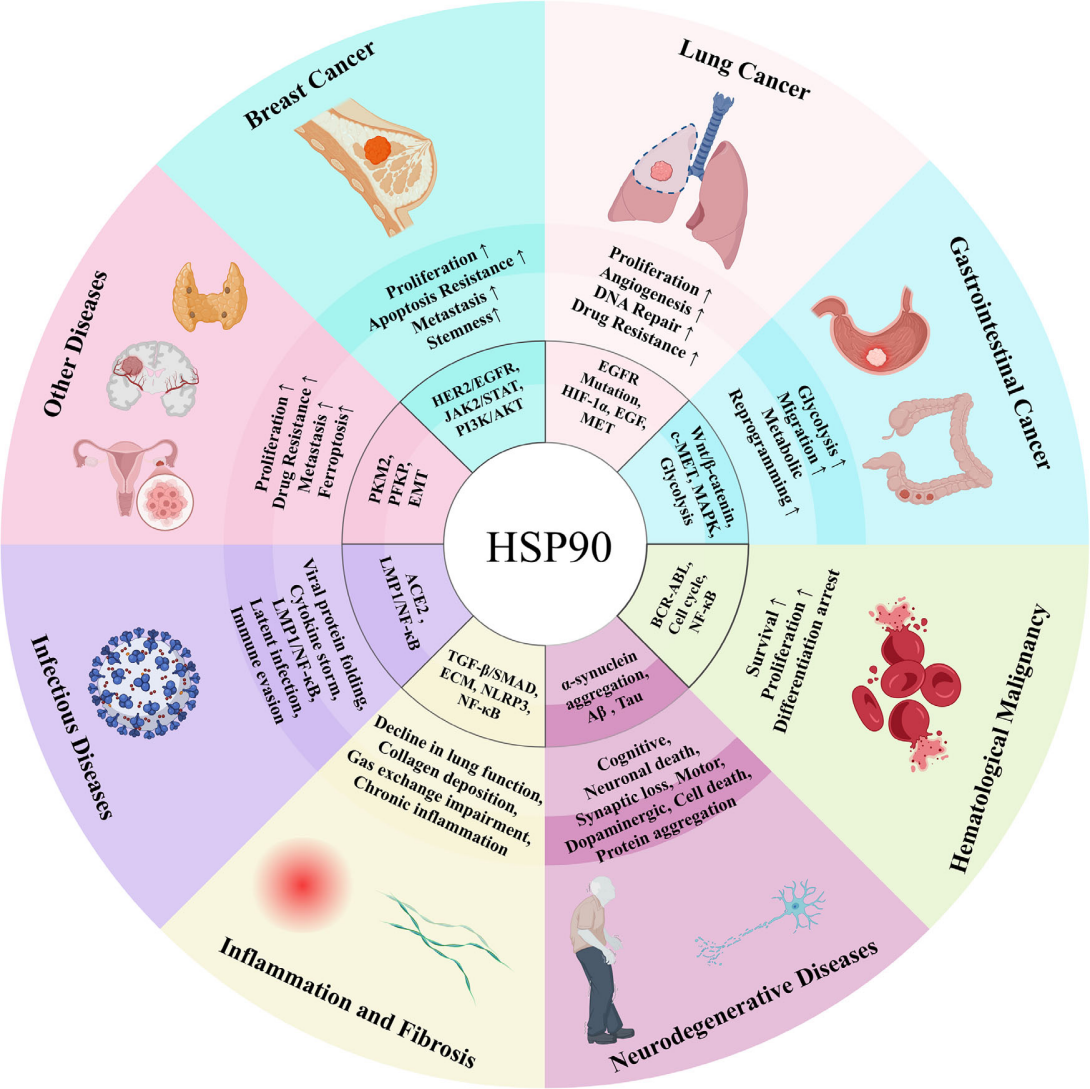
HSP90‐associated signaling pathways and cellular functions in different diseases.

In summary, HSP90 serves as a key molecular chaperone in multiple malignant tumors‐where its increased expression stabilizes numerous oncoproteins‐including EGFR, HER2, and HIF‐1α‐enabling cancer cells to sustain hyperactive growth, survival, and metastatic capabilities, a phenomenon often described as “oncogene addiction”. As depicted in Figure 3, elevated HSP90 levels associated with aberrant signaling functions across diverse disease types are commonly linked to disease progression as well as poor clinical outcomes, establishing its relevance as both a biomarker and critical therapeutic or intervention target. Targeting HSP90 with specific inhibitors disrupts these client proteins and signaling pathways, elicits robust anti‐tumor responses, enhances the efficacy of conventional therapies, and helps overcome treatment resistance, underscoring its utility as a versatile strategy in therapy.

## 4 Development of Chemical Therapeutics Targeting HSP90

**FIGURE 4:**
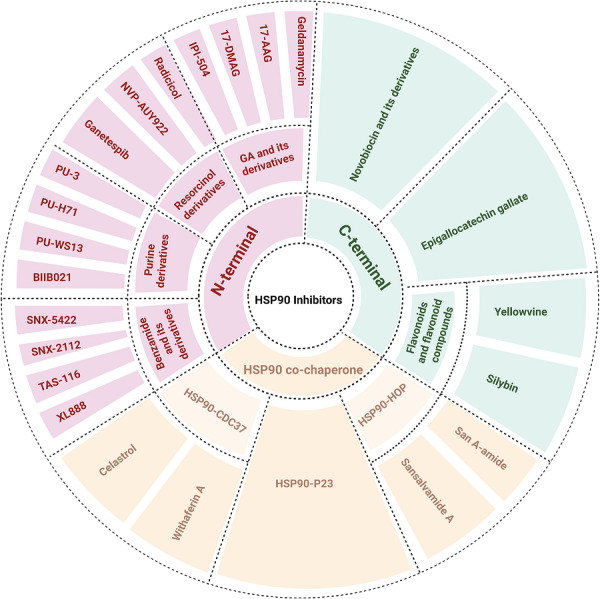
Classification of HSP90 chemical inhibitors and their representative compounds.

As mentioned above, HSP90 activity is markedly elevated across a broad range of human malignancies. Given HSP90's central role in stabilizing numerous oncoproteins, including EGFR and c‐Met, blocking its chaperone function has become a compelling approach in anti‐cancer drug development [93]. HSP90‐targeted therapy, central to rational drug design, employs innovative inhibitors that precisely interfere with its interaction with key client proteins. By disrupting these interactions, oncogenic clients undergo proteasomal degradation, effectively impairing tumor cell growth and survival. As shown in Figure 4, HSP90 inhibitors and related representative compounds are systematically categorized by key binding sites (N‐terminal and C‐terminal) and interactions with HSP90 co‐chaperones. This section will outline them and discuss their therapeutic potential and clinical efficacy as follows.

### 4.1 N‐Terminal (NT) HSP90 Inhibitors

#### 4.1.1 Geldanamycin and Its Derivatives

**Geldanamycin (GA)**, a benzoquinone‐based ansamycin antibiotic, is a potent and highly selective inhibitor of HSP90 that targets its NT ATP‐binding pocket. Crystallographic analyses of the HSP90‐GA complex reveal that GA acts as an ATP‐competitive inhibitor, occupying the NTD as well as inducing conformational modifications that block the chaperone's ATPase activity [94]. Although GA exhibits marked anti‐tumor activity in laboratory models, its clinical translation has been hindered by poor solubility, limited stability in vivo, as well as notable hepatotoxicity [95]. These drawbacks have driven substantial structural optimization efforts, leading to the creation of several semi‐synthetic derivatives with enhanced pharmacological characteristics and greater therapeutic promise.

**17‐Allylamino‐17‐demethoxygeldanamycin (17‐AAG)**, a GA derivative in which the 17‐methoxy group is substituted with an allylamino moiety, exhibits enhanced biological activity and a more favorable toxicity profile than the parent compound [96]. 17‐AAG also potently downregulates cell surface ERBB2 [97]. Nevertheless, challenges like poor solubility and potential off‐target toxicity have constrained its performance in advanced tumors, underscoring the need for further optimization.

**17‐dimethylaminoethylamino‐17‐desmethoxygeldanamycin (17‐DMAG)**, a next‐generation HSP90 inhibitor, exhibits markedly higher aqueous solubility as well as oral bioavailability due to its ionizable amino groups, resulting in stronger antitumor activity compared with 17‐AAG [98]. Clearly, compared to 17‐AAG, the presence of ionizable amino groups in 17‐DMAG enhances its water solubility, improves oral bioavailability, and exhibits stronger antitumor activity. The compound further induces dose‐dependent growth suppression, G2/M cell cycle arrest, as well as apoptosis in hormone‐responsive BC models [99]. Consistent with these findings, 17‐DMAG treatment in a mouse leukemia model markedly reduces white blood cell counts and extends survival [98].

**IPI‐504,** a hydroquinone hydrochloride 17‐AAG derivative, was engineered to address the limited solubility of its parent compound and shows markedly better aqueous compatibility and improved target affinity. In patients with non‐small cell lung cancer (NSCLC), particularly those harboring ALK rearrangements, IPI‐504 demonstrated notable therapeutic activity [100]. Although early laboratory studies and initial clinical trials were encouraging, subsequent Phase I‐III investigations in MM, BC, and GIST did not achieve their primary efficacy endpoints [101]. These trials reported either minimal anti‐tumor effects or notable safety concerns, including treatment‐related deaths, ultimately leading to the termination of its clinical development.

#### 4.1.2 Resorcinol Derivatives

**Radicicol (RD)** is a macrolide antibiotic characterized by its central resorcinol moiety [94]. Despite its inherent chemical instability and poor in vivo efficacy, RD has played an influential role in guiding the design of modern HSP90 inhibitors such as ganetespib [94]. These compounds, while not direct derivatives of RD, contain a shared resorcinol core. They inhibit HSP90's chaperone function like RD by targeting the ATP‐binding site. The consistent presence of the resorcinol pharmacophore is essential for the inhibitory action of these compounds against HSP90.

**NVP‐AUY922**, a second‐generation isoxazole‐resorcinol HSP90 inhibitor, suppresses chaperone function by blocking the ATPase activity of HSP90. In mice undergoing allogeneic hematopoietic stem cell (HSC) transfer, NVP‐AUY922 induced the degradation of the epigenetic regulator Ezh2 in T cells, destabilizing the protein without interfering with the overall transplant regimen [102]. NVP‐AUY922 synergistically enhances cell death and decreases VEGF in MCF‐7 BC cells [103]. In leukemia cells, it induces a dose‐dependent rise in HSP70 and depletes HSP90 client proteins, crucial for cell cycle and survival, like phosphorylated AKT and IKKs [104]. The HSP90 inhibitor AUY922 significantly suppressed the growth of nasopharyngeal carcinoma (NPC) and NSCLC cells, including cisplatin‐resistant subtypes, in both in vitro as well as in vivo models, suggesting its potential to reverse cisplatin resistance [105]. Based on its potent mechanism of action, NVP‐AUY922 has been evaluated in relapsed or refractory MM, NSCLC, and HER2‐positive or ER‐positive metastatic BC [106].

**Ganetespib**, a second‐generation HSP90 inhibitor, exhibits broad anti‐tumor activity [107]. In Squamous Cell Carcinoma of the Head and Neck (HNSCC), it reduces glycolytic flux by inhibiting key enzymes like PKM2 and PFKP, thereby downregulating tumor‐associated glycolysis [108]. In a Phase I trial involving patients with HER2‐positive advanced BC, the combination of ganetespib with low‐dose paclitaxel and trastuzumab produced a robust triplet therapy effect, leading to a recommended Phase II dose of 150 mg/m^2^ [64]. However, in a Phase II study involving patients with advanced pancreatic cancer, the trial was terminated early because ganetespib, when used alone, did not demonstrate adequate anti‐tumor activity, even though treatment‐related toxicities remained well controlled [109]. These findings offer critical insights for guiding the continued therapeutic development of ganetespib.

#### 4.1.3 Purine Derivatives

**PU‐3**, the first synthetic HSP90 inhibitor based on a purine scaffold, was designed using structure‐based drug discovery approaches that specifically target the unique bergerat fold within the NT ATP‐binding pocket of HSP90 [110]. Its design leveraged insights from the conformational dynamics associated with ATP/ADP binding, as well as the structural features of the GHKL‐type ATPase domain.

**PU‐H71**, a purine‐scaffold HSP90 inhibitor derived from PU‐3, exhibits enhanced aqueous solubility and strong binding specificity for the ATP‐binding pocket of HSP90 [111]. Preclinical studies demonstrate that PU‐H71 potently inhibits HSP90 chaperone function, leading to the destabilization of B‐cell receptor (BCR) kinases and subsequent induction of mitochondrial‐mediated apoptosis in CLL cells [112]. Notably, this study identified PU‐H71‐based positron emission tomography (PU‐PET) as a promising non‐invasive biomarker for predicting treatment response [113].

**PU‐WS13** is a selectively designed GRP94 inhibitor that competitively occupies the ATP‐binding pocket of GRP94 and blocks its ATPase activity [114]. This interaction exhibits high selectivity, achieving a semi‐maximal effective concentration for GRP94 inhibition, while displaying markedly lower activity toward other HSP90 family members‐HSP90α, HSP90β, and TRAP1 [115]. This selectivity allows for a precise study of GRP94 functions while minimizing off‐target effects. In HER2‐overexpressing BC cell lines, PU‐WS13 disrupts the GRP94‐mediated circular structure of HER2 proteins localized on the plasma membrane, thereby inducing apoptosis as well as reducing cancer cell viability [115]. PU‐WS13 shows significant efficacy and high safety in RTK‐overexpressing cancers and provides an intervenable target for chaperone‐mediated pathological protein assembly by targeting aberrant N‐glycosylation to regulate GRP94 functional transition [116]. PU‐WS13 demonstrated potent anti‐cancer efficacy by suppressing the proliferation of both leukemic and solid tumor cells, while significantly downregulating PD‐L1 expression on cancer cell surfaces [117].

**BIIB021**, the first orally bioavailable synthetic HSP90 inhibitor structurally distinct from GA, exerts its anti‐tumor effects by simultaneously suppressing canonical as well as non‐canonical NF‐κB signaling pathways, ultimately inducing G0/G1 phase cell cycle arrest and apoptosis in relevant cell lines [118]. As the pioneering purine‐based HSP90 inhibitor to advance into clinical evaluation, BIIB021 demonstrated promising efficacy in a Phase II trial involving patients with GIST. However, subsequent development was terminated following a strategic decision to discontinue the oncology research program [119].

#### 4.1.4 Benzamide and Its Derivatives

Benzamide‐derived HSP90 inhibitors feature a core structure that competitively binding to the NT ATP pocket, disrupting chaperone function. Agents such as SNX‐5422, TAS‐116, and XL888 selectively target HSP90, induce oncogenic client protein degradation, and demonstrate antitumor efficacy in preclinical and clinical studies.

**SNX‐5422**, an orally bioavailable HSP90 inhibitor, exhibits enhanced water solubility and high specificity for the ATP‐binding pocket of HSP90, demonstrating promising therapeutic potential in preclinical studies [120]. SNX‐5422 showed good tolerability and reached target inhibition levels in Phase I trials for advanced solid tumors and lymphomas. Its development was halted due to dose‐limiting ocular toxicities, including the risk of irreversible retinal damage [121].

**SNX‐2112** is a potent and selective synthetic inhibitor that exerts broad‐spectrum antitumor activity both in vitro and in vivo by inducing the degradation of multiple HSP90 client proteins, thereby disrupting critical oncogenic signaling pathways and inducing cell cycle arrest as well as apoptosis [122]. SNX‐5542, an orally bioavailable SNX‐2112 prodrug, demonstrates favorable pharmacokinetics with enhanced tumor accumulation and sustained target inhibition, showing significant efficacy in HER2‐positive and MET‐amplified xenograft models. It overcomes MET resistance in preclinical studies and exhibits antiangiogenic and anti‐osteoclastogenic effects in the tumor microenvironment, warranting further investigation [123].

**TAS‐116** is a highly selective oral inhibitor that preferentially targets the cytosolic isoforms HSP90α as well as HSP90β, demonstrating minimal affinity for the ER paralog GRP94 and mitochondrial isoform TRAP1 within the HSP90 family [124]. In cervical cancer models, oral TAS‐116 demonstrates potent antitumor activity in xenografts without causing significant body weight loss, indicating an improved safety profile relative to earlier pan‐inhibitors [125]. In a Phase II trial, TAS‐116 monotherapy demonstrated efficacy in GIST patients resistant to standard therapy, with a prolonged progression‐free survival, warranting further development [126].

**XL888** is a potent ATP‐competitive inhibitor of HSP90 that effectively suppresses the proliferation of a broad spectrum of human tumor cell lines by inducing the degradation of key oncogenic client proteins, including HER2, MET, mutant BRAF, and EGFR [127].

### 4.2 C‐terminal (CT) HSP90 Inhibitor

#### 4.2.1 Novobiocin and Its Derivatives

The earliest identified inhibitors targeting the CTD of HSP90 were novobiocin and coumermycin A1. However, due to the relatively weak inhibitory activity of novobiocin against HSP90, subsequent structure‐activity relationship studies guided the rational design of optimized analogs, including A4 and KU‐32. These derivatives were engineered to structurally mimic adenine and guanine nucleotides, providing specific hydrogen bond acceptors and donors that enhance binding affinity and specificity for the CT nucleotide‐binding pocket [128].

#### 4.2.2 Epigallocatechin Gallate (EGCG)

Like novobiocin, EGCG functions as an HSP90 inhibitor by selectively targeting the CTD. This interaction modulates HSP90 conformation, impairing its chaperone function and dimerization capacity, which are essential for client protein maturation [129]. In gastrointestinal tumors, EGCG inhibits HSP90, leading to the disruption of the JAK‐STAT signaling pathway, suppression of STAT3 nuclear translocation, as well as decreased DNA‐binding activity [130]. Consequently, EGCG treatment downregulates key HSP90‐dependent oncoproteins [131]. EGCG impedes angiogenesis in vitro by inhibiting IL‐6‐induced endothelial cell proliferation, tubulogenesis, and angiogenic processes, demonstrating its multifaceted therapeutic potential in oncology [132]. To enhance its delivery and efficacy, EGCG has been incorporated into stimuli‐responsive nanoplatforms, such as M/D@P/E‐P, which enables synergistic gas therapy and photothermal therapy [133]. The EGCG‐MnCO complex with near‐infrared light, inducing photothermal destruction and controlled release, overcomes tumor thermos‐resistance by impairing mitochondrial function and promoting apoptosis [133].

#### 4.2.3 Flavonoids and Flavonoid Compounds

**Yellowvine**, a naturally occurring flavonoid, demonstrates anti‐proliferative and antiangiogenic activities in tumor models. These effects result from its interaction with the CTD of HSP90, leading to disruption of chaperone function [128]. **Silybin**, a traditional flavonoid derived from milk thistle fruit, exhibits anti‐cancer activity across multiple cancer cell lines [134]. Its mechanism involves binding to the CTD of HSP90, promoting degradation of HSP90‐dependent client proteins without altering total HSP90 expression.

### 4.3 Inhibitors That Interfere With HSP90 Co‐Chaperone Binding

HSP90's ATP‐driven conformational cycle, regulated by nucleotide binding and hydrolysis, enables it to function within a chaperone network. By forming transient complexes with co‐chaperones, HSP90 stabilizes, matures, and activates client proteins while preventing their degradation. The client‐specific mechanisms mediated by different co‐chaperones offer opportunities for targeted HSP90 inhibition through selective disruption of these interactions, potentially improving selectivity and reducing toxicity compared to pan‐HSP90 inhibition strategies.

#### 4.3.1 HSP90‐CDC37 Interaction Inhibitor

CDC37 regulates cell division by facilitating the recruitment of protein kinases to HSP90, a critical step for maintaining the stability as well as functional integrity of the HSP90‐kinase chaperone complex [135]. The CDC37‐HSP90 interaction encompasses a substantial binding interface, through which CDC37 modulates HSP90's ATPase cycle by preventing closure of the NT nucleotide‐binding pocket lid, thereby inhibiting ATP hydrolysis. This regulatory mechanism ensures proper client kinase folding and provides avenues for synergistic inhibitory effects when combined with other therapeutic strategies [136].

**Celastrol**, a pentacyclic triterpenoid extracted from medicinal plants, exhibits a broad spectrum of biological activities. It functions as a potent activator of HSF1, promoting transcriptional upregulation of HSPs, which underlies its antiproliferative and neuroprotective effects [137]. Notably, celastrol covalently modifies specific cysteine residues on CDC37, interfering with its interaction with HSP90 [138]. This disruption promotes the ubiquitin‐proteasome‐mediated degradation of HSP90‐dependent client proteins. Additionally, celastrol induces amyloid‐like fibrillization of the co‐chaperone P23, inactivating its function and selectively destabilizing steroid hormone receptors [139]. **Withaferin A**, a steroidal lactone, demonstrates antiproliferative and antiangiogenic activities. It exerts concentration‐dependent destabilization of key HSP90 client proteins, including AKT and CDK4, etc. [140]. Co‐immunoprecipitation assays confirm that Withaferin A disrupts the HSP90‐CDC37 complex, impairing chaperone complex assembly, as further validated by molecular docking studies [141].

#### 4.3.2 HSP90‐P23 Interaction Inhibitor

The small acidic protein P23 interacts with the NT and intermediate domains of HSP90 and is necessary for stabilizing steroid hormone receptors [142]. Gedunin, a neem tree compound, has long been used against malaria and infections. Studies suggest its anti‐tumor properties, targeting prostate, colon, and ovarian cancer cells. It promotes the breakdown of HSP90 client proteins, including HER2 and Raf‐1 [143]. Molecular docking and mutagenesis studies suggest that gedunin directly interacts with P23, disrupting the HSP90‐P23 interaction and impairing client protein binding [144].

#### 4.3.3 HSP90‐HOP Interaction Inhibitor

HSP90's CTD terminates in a highly conserved MEEVD motif, which functions as a critical binding site for co‐chaperones containing tetratricopeptide repeat (TPR) domains. The HOP utilizes its TPR domains to facilitate functional coordination between HSP70 and HSP90 [145].

**Sansalvamide A (San A),** a cyclic depsipeptide derived from marine fungi, demonstrates moderate antitumor activity [146]. As a depsipeptide, it contains ester bonds that are prone to ring‐opening degradation by esterases, resulting in poor in vivo stability, which has motivated subsequent structural optimization [147]. **San A‐amide**, a derivative in which the labile ester bond is replaced with a stable amide bond, exhibits a tenfold increase in potency compared to the parent compound [148]. San A‐amide binds to HSP90's NTD‐MD, allosterically disrupting HOP's interaction with HSP90, rather than inhibiting NT ATPase activity [149]. Over the past decades, numerous San A‐amide analogs have been developed, showing potent and broad‐spectrum cytotoxicity against pancreatic, breast, prostate, and colorectal carcinomas.

However, the clinical translation of these HSP90 inhibitors still faces multiple challenges. Firstly, pharmacokinetic limitations and off‐target toxicity pose major obstacles. These compounds are difficult to apply clinically due to poor solubility, low stability, and hepatotoxicity. Although SNX‐5422 demonstrated potent targeted inhibition, its development was terminated due to dose‐limiting ocular toxicity (including risks of irreversible retinal damage). Next, tumor cells can re‐activate escape pathways such as RAF/MEK/ERK and PI3K/AKT/mTOR after HSP90 inhibition, or maintain oxidative phosphorylation through metabolic plasticity in hypoxic regions dependent on mitochondrial homologous protein TRAP1, thereby circumventing HSP90 blockade effects. The subsequent activation of the bypass signaling pathways further compromises the efficacy of monotherapies, thereby underscoring the need for a deeper understanding of the underlying pathological mechanisms and the development of novel therapeutic strategies.

## 5 HSP90 Mediated Tumor Recurrence and Drug Resistance

Despite the availability of these inhibitors, clinical efficacy is frequently limited by adaptive resistance, which leads to tumor recurrence. Recurrence, often manifesting as relapse after seemingly successful initial treatment, represents a major clinical challenge and is a primary driver of cancer‐related mortality [150]. This phenomenon is frequently driven by a subset of cancer cells that evade therapy, enter a dormant state, or acquire resistance mechanisms, later re‐emerging as recurrent disease [150]. At present, single‐agent HSP90 inhibition rarely yields deep or durable responses and points toward rational strategies to enhance efficacy. Co‐targeting HSP90 can blunt the compensatory HSR; combining HSP90 inhibitors with other agents like kinase inhibitors, or DNA damage response inhibitors can exploit reprogrammed survival dependencies [151], and incorporating autophagy inhibitors can prevent cytoprotective clearance of damaged clients [152]. In parallel, nanoparticle‐ and antibody‐based delivery systems that circumvent drug efflux and concentrate HSP90 inhibitors within tumors offer a way to overcome pharmacokinetic resistance and widen the therapeutic window.

Clearly, HSP90 plays a critical, though often indirect, role in promoting tumor recurrence through several interconnected mechanisms, primarily by fostering cancer stem cell (CSC) plasticity, supporting the persistence of dormant cells, as well as modulating the TME [153]. In the following sections, these mechanistic principles can be mapped onto specific disease contexts and combination regimens, highlighting how understanding resistance to HSP90 inhibition informs the design of next‐generation therapies that better exploit the chaperone addiction of malignant cells while anticipating and forestalling adaptive escape.

### 5.1 Cancer Stem Cell State and Plasticity

One key mechanism involves the contribution of HSP90 to CSC properties and tumor plasticity. CSCs, marked by self‐renewal capacity, differentiation potential, as well as inherent therapy resistance, are key drivers of tumor initiation, metastasis, and recurrence [154]. HSP90 and its co‐chaperones also support cell‐state plasticity and CSC phenotypes, which are intrinsically more drug‐resistant [155]. HSP90 activity buffers the phenotypic consequences of oncogenic mutations, enabling tumors to explore a broader range of adaptive states under therapy. CSC‐enriched populations frequently display elevated HSP expression, enhanced DNA repair, and robust anti‐apoptotic signaling, making them less susceptible to HSP90 inhibition and standard‐of‐care therapies [156]. In chronic myeloid leukemia (CML) models, combined inhibition of HSP90 and SIRT1 more effectively targets stem‐like, therapy‐refractory cells than either agent alone [155]. The TME further modulates resistance, hypoxia, low pH, nutrient stress, and inflammatory cytokines all drive HSP90 expression and activity, reinforcing a protective niche for tumor cells exposed to chemotherapy or targeted agents [157]. HSP90 in stromal cells and endothelial cells can also support angiogenesis, invasion, and immune evasion, indirectly reducing the impact of HSP90‐directed therapies on overall tumor fitness [158]. Thus, resistance should be considered not only as a tumor cell‐intrinsic problem, but also as a product of dynamic crosstalk between cancer cells and their microenvironment. HSP90 stabilizes numerous signaling proteins and transcription factors that maintain the stem‐like phenotype. This is evidenced by studies showing that HSP90 inhibitors can impair cancer stem‐like traits in HER2‐positive BC and target both CSCs and non‐CSCs in NSCLC [63]. The novel CT HSP90 inhibitor NCT‐80 disrupted the HSP90‐STAT3 interaction, degraded STAT3, and potentiated chemotherapy effects by targeting CSC as well as non‐CSC populations in NSCLC [159]. The ability of cancer cells to undergo lineage plasticity‐transitioning between states like epithelial‐to‐mesenchymal transition (EMT) or neuroendocrine differentiation, is another critical mechanism of treatment resistance and recurrence, particularly in PCa [160]. HSP90's broad chaperone activity likely stabilizes proteins critical for these plastic transitions, thereby enhancing the adaptability and resilience of cancer cells that contribute to recurrence.

### 5.2 Sustenance of Dormant Cancer Cells

Furthermore, HSP90 contributes to the persistence as well as reactivation of quiescent or dormant cancer cells (DCCs). These non‐proliferating cells evade detection and are highly resistant to therapies targeting rapidly dividing cells, serving as a reservoir for future relapse [150, 161]. Although direct evidence linking HSP90 to DCC maintenance is still emerging, its role in stabilizing essential survival proteins and stress response pathways suggests it is crucial for the long‐term viability of these dormant populations. By preserving proteins that enable cells to withstand stress and survive under low‐metabolic conditions, HSP90 indirectly supports the persistence of DCCs, which can later re‐enter the cell cycle and contribute to recurrence.

### 5.3 Modulation of the Tumor Microenvironment

HSP90 also significantly influences the TME in ways that promote recurrence. HSP90 can modulate the TME in several ways; for example, extracellular HSP90 can promote M2 polarization of macrophages, creating an immunosuppressive, protumorigenic environment that facilitates tumor growth and potential recurrence [162]. Moreover, HSP90 inhibitors attenuate the immunosuppressive function of regulatory T cells (Treg), which are key suppressors of antitumor immunity, suggesting that HSP90 inhibition could enhance immune responses and potentially prevent recurrence [163]. Interactions between tumor cells and cancer‐associated fibroblasts (CAFs), often mediated by HSP90‐stabilized growth factors, can also create conditions conducive to recurrence [164]. Neoadjuvant therapy in HCC patients showed increased immune cells and CAFs in responding tumors. Despite a significant treatment response, tumor recurrence still frequently occurred in a subset of patients. This indicates the presence and potential involvement of tumor heterogeneity, immune evasion, and CSCs, which may collectively drive tumor recurrence [165].

HSP90's pervasive role in stabilizing oncoproteins, maintaining CSC characteristics, supporting dormant cell survival, and modulating the TME collectively positions it as a critical driver of tumor recurrence. HSP90 inhibitors are particularly promising for overcoming diverse forms of drug resistance due to their capacity to simultaneously target multiple oncogenic pathways, offering a distinct advantage over single‐pathway therapies. A detailed understanding of these intricate mechanisms is crucial for devising strategies that not only eliminate primary tumors but also prevent disease recurrence. To illustrate these strategies, the following cases exemplify the use of HSP90‐targeted therapies to overcome drug resistance, detailing the scientific basis for each.

**FIGURE 5:**
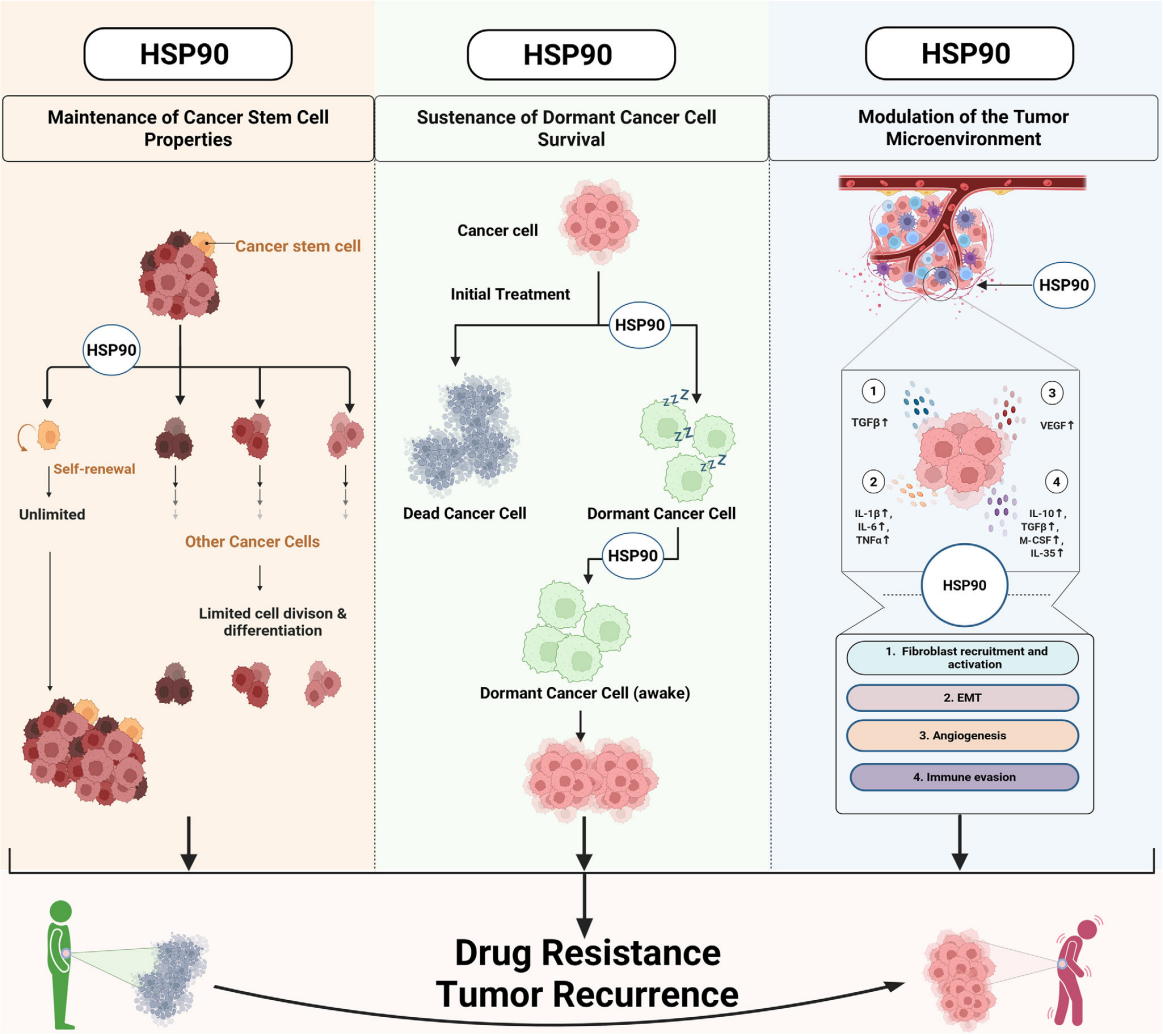
HSP90‐mediated mechanisms of tumor recurrence and drug resistance.

As summarized in Figure 5, HSP90 is deeply implicated in mechanisms underlying tumor relapse, recurrence, and resistance to therapy across multifaceted roles in multiple cancer types. In conclusion, HSP90 inhibitors are emerging as powerful anti‐cancer agents due to their ability to concurrently modulate multiple oncogenic pathways. Their demonstrated efficacy to counter resistance to conventional chemotherapy as well as targeted therapies‐combined with their capacity to trigger apoptosis, halt cell cycle progression, and exert senolytic effects‐highlights their potential as integral contributors to the next generation of cancer treatment. The ongoing development of more specific and less toxic inhibitors, particularly those targeting specific isoforms or co‐chaperone interactions, further enhances their potential to overcome the persistent challenge of therapeutic resistance.

## 6 HSP90 Derived Novel Drugs, Delivery Strategies, and Combination Therapies

HSP90 is a critical molecular chaperone that stabilizes numerous oncoproteins, making it an attractive target in cancer therapy. However, the clinical efficacy of HSP90 inhibitors as monotherapies has been limited by several adaptive resistance mechanisms. For example, a key challenge is the HSR, where inhibitor‐induced stress triggers HSF1‐mediated upregulation of compensatory chaperones, sustaining tumor survival [16]. Furthermore, cancer cells exhibit metabolic plasticity; for instance, dormant cells in hypoxic tumor cores may switch their energy reliance between glycolysis (often supported by cytosolic HSP90) and oxidative phosphorylation (OXPHOS, regulated by the mitochondrial homolog TRAP1) [166]. Additionally, resistance can arise from the reactivation of bypass signaling pathways, such as RAF/MEK/ERK and PI3K/AKT/mTOR, upon HSP90 inhibition [167]. To address these limitations, novel HSP‐associated drug forms, delivery systems, and rational combination therapies are being explored. Strategies include combining HSP90 inhibitors with drugs that target these escape pathways, such as CDK4/6 inhibitors (which can interrupt the HSF1‐HSP90 axis independently of P53 status) or MEK/ERK inhibitors, to achieve synergistic effects and prevent tumor adaptation, which represents a promising approach to enhance efficacy and overcome resistance [16, 167]. This has led to a strategic pivot toward exploring combination therapies that leverage synergistic actions and target specific resistance pathways to achieve better outcomes.

### 6.1 PROTACs

Proteolysis‐Targeting Chimeras (PROTAC) technology is gaining attention as an effective method in oncology due to its broad target scope, high specificity, low toxicity, and reduced susceptibility to drug resistance. The HSP90‐directed PROTAC molecule BP3 effectively induces HSP90 degradation and restricts the growth of human BC cells [168]. Furthermore, the rational design of novel HSP90 inhibitors is being accelerated through computational simulations and ligand‐based optimization approaches. Based on its distinct structural and functional characteristics, numerous conventional inhibitors have been designed to target various domains and co‐complexes of HSP90. These representative agents of HSP90‐associated novel forms are systematically compared in Table 1.

**TABLE 1:** The advantages and disadvantages of representative classes of HSP90 inhibitors.

Inhibitor class	Examples	Advantages	Disadvantages/Challenges
N‐Terminal Inhibitors	Ganetespib, 17‐AAG, AUY922	Potent, broad client degradation, established mechanism.	High systemic toxicity, primary mechanism for triggering the cytoprotective Heat Shock Response (HSR) via HSF1 activation.
C‐Terminal Inhibitors	EGCG	Reduced Heat Shock Response (HSR) induction, potentially lower toxicity, targets dimerization.	Less established clinical data, mechanism of action may differ from NTD inhibitors, requiring careful validation
Isoform‐Selective Inhibitors	Pimitespib (TAS‐116)	Improved selectivity, reduced off‐target effects, successfully marketed.	Potential for residual oncogenic activity if non‐targeted isoforms compensate.
Dual/Multi‐Target Inhibitors	HDAC6/HSP90 dual inhibitors	Synergistic effects, targeting multiple resistance pathways simultaneously, potential for immunomodulation.	Complex pharmacokinetics, risk of increased toxicity if targets are non‐selective.
PROTACs and Targeted Delivery	BP3@HSA NPs, A6‐NPs	Highly efficient degradation of specific proteins (even non‐clients like PARP1), reduced systemic toxicity, enhanced tumor targeting.	Development complexity, stability challenges, reliance on specific E3 ligases.

### 6.2 Novel Drug Delivery Systems and Multi‐Functional Approaches

To address limitations such as poor pharmacokinetics, off‐target toxicity, as well as drug resistance, innovative drug delivery systems and multi‐functional nanoplatforms are being developed for HSP90 inhibitors [21, 133]. Nano‐carriers can improve drug solubility, promote tumor‐targeted accumulation via the enhanced permeability and retention effect, as well as enable controlled drug release, thereby increasing treatment efficacy while reducing systemic toxicity. Examples include stimulus‐responsive nanoplatforms for synergistic gas and photothermal therapy (PTT) with HSP90 inhibitors, and metal‐enriched nano inhibitors for overcoming heat resistance in hyperthermia [169]. For example, the multi‐stimuli responsive theranostic nanoplatform M/D@P/E‐P, achieves synergy between gas therapy and photothermal therapy: carbon monoxide impairs mitochondrial function to indirectly inhibit HSP90, while EGCG directly inhibits HSP90, collectively overcoming tumor thermos‐resistance and significantly enhancing photothermal therapy efficacy under near‐infrared (NIR)/pH dual‐stimuli response [133]. Another nano‐platform, GI‐MPDA@BSA, employs a triple synergistic mechanism of “mild PTT+ chemotherapy+HSP90 inhibition”, improving the therapeutic effect on TNBC while reducing damage to normal tissues and potentially activating systemic antitumor immunity [170]. Further research is needed to optimize the design of these nano‐carriers for efficient tumor penetration, controlled release, and effective modulation of the TME [171]. Representative novel drug delivery systems for HSP90 inhibitors are compared in Table 2.

**TABLE 2:** The comparison of different novel drug delivery systems for HSP90 inhibitors.

Delivery System	Core Idea	Advantages	Disadvantages	Representative reference
Hollow mesoporous nanoparticles (e.g., GI‐MPDA@BSA)	Encapsulation of HSP90 i within porous nanoparticles for controlled release and synergistic therapy.	Enhanced tumor accumulation (EPR effect), reduced systemic toxicity, combination with photothermal therapy, immune response elicitation.	Potential for complex synthesis, biocompatibility concerns, scaling up production.	Yuan et al. [170]
Stimuli‐responsive theranostic Nanoplatforms (e.g., M/D@P/E‐P, G@P/TGSAs)	Nanocarriers designed to release drugs in response to tumor‐specific stimuli (pH, ROS, NIR light) and offer diagnostic capabilities.	Targeted drug release, multi‐modal therapy (e.g., gas therapy, PTT, PDT), reduced thermo‐resistance, imaging capabilities.	Complexity of design and synthesis, potential for premature release, long‐term stability.	Yang et al. [133]
PROTAC‐Nanocarriers (e.g., Albumin encapsulated BP3)	Encapsulation of HSP90‐targeting PROTACs within nanoparticles to improve delivery and stability.	Enhanced anti‐tumor activity, improved pharmacokinetic properties, targeted protein degradation, overcoming PROTAC delivery challenges.	Specificity of PROTACs, potential for off‐target degradation, stability within nanocarrier.	Liu et al. [168]
Metal‐enriched nano‐inhibitors (e.g., Mn‐EGCG)	Bifunctional nano‐inhibitors formed by metal ions and HSP90 i for synergistic effects and overcoming heat resistance.	Enhanced oxidative stress, pyroptosis induction, “cold” to “hot” tumor conversion, immune response stimulation.	Potential metal toxicity, precise control over ion release, complex interactions.	Wang et al. [169]

### 6.3 Combination Therapies With HSP90 Inhibitors

#### 6.3.1 Combination With Conventional Chemotherapy

HSP90 inhibitors like onalespib boost cancer cell susceptibility to standard chemotherapy by disrupting common survival mechanisms. This approach showed antitumor effects in advanced TNBC patients, including taxane‐pretreated individuals, when combined with paclitaxel, while preserving a manageable safety profile [172, 173]. However, synergistic effects are not universal; an antagonistic interaction was observed between the HSP90 inhibitor XL‐888 and 5‐fluorouracil in BC models, underscoring the necessity of carefully evaluating drug interactions [174]. Conversely, novel HSP90‐targeting PROTACs exhibit enhanced synergy with cisplatin in cervical cancer, indicating that the specific class and mechanism of the HSP90 inhibitor critically influence combination outcomes [125].

#### 6.3.2 Combination With Targeted Agents

HSP90 inhibitors are particularly effective when combined with other targeted therapies, as they promote the degradation of numerous oncogenic client proteins. **Histone deacetylase (HDAC) Inhibitors**: Combining HDAC inhibitors with HSP90/TRAP1‐targeting agents produces strong synergistic effects in TNBC models, effectively downregulating hypoxia‐related genes and inhibiting cell migration more potently than monotherapies [175]. Dual HDAC and HSP90 inhibitors identified via in silico modeling offer a promising strategy for metastatic TNBC [53]. **PARP Inhibitors**: HSP90 inhibitors like pimitespib can enhance the potency of PARP inhibitors in insensitive BC cells by impairing the homologous recombination repair pathway, leading to increased DNA damage and antitumor activity [176]. A Phase 1 study combining pimitespib with the PARP inhibitor niraparib in solid tumors, including BC, showed manageable safety and potential efficacy, particularly in BRCA‐associated cancers resistant to PARP inhibitors [177]. **CDK4/6 Inhibitors**: This Phase Ib trial assesses combining palbociclib with TAS‐116 in advanced BC patients resistant to prior CDK4/6 inhibitors to evaluate safety and overcome treatment resistance [178]. **HER2‐Targeted Therapies**: In HER2‐positive BC, HSP90 inhibitors can overcome trastuzumab resistance. Novel CT inhibitors such as NCT‐547 and HVH‐2930 induce apoptosis and promote the degradation of HER2 family members [63]. HSP90 is upregulated after HER2 downregulation, enhancing trastuzumab and docetaxel's growth‐inhibiting effects, hinting at a potential link between high HSP90 levels and improved progression‐free survival with the triple therapy [62]. **AKT/mTOR Inhibitors**: Combining HSP90 inhibitors with mTORC1/2 inhibitors markedly reduces BC cell proliferation, migration, as well as invasion by downregulating AKT phosphorylation and disrupting F‐actin organization [179]. **HSF1 Inhibitors**: Since HSP90 inhibition can activate the HSF1 resistance pathway, combining HSP90 inhibitors with HSF1 inhibitors (e.g., KRIBB11) shows synergistic effects in BC models, enhancing efficacy in cell viability and clonogenic assays [180].

#### 6.3.3 Combination With Physical Therapies

HSP90 inhibitors can potentiate the potency of physical anti‐cancer modalities. **Photothermal/Photodynamic Therapy (PTT/PDT)**: HSP90 inhibitors within hollow mesoporous nanoparticles boost the combo of photothermal and chemotherapy for TNBC, inhibiting tumor growth and activating immune response with laser light [170]. Similarly, SNX2112‐encapsulated nano‐micelles suppress the HSP90‐HIF1α pathway, improving combined photothermal and photodynamic therapy for TNBC [58]. **Microwave Thermal Therapy (MWTT)**: Hydrogen sulfide‐mediated gas therapy combined with HSP90 downregulation synergistically enhances MWTT by inhibiting ATP production and promoting apoptosis, thereby reducing risks of tumor recurrence and metastasis [181]. **Hyperthermic Intraperitoneal Chemotherapy (HIPEC)**: Metal‐enriched HSP90 nano‐inhibitors have been designed to overcome thermal resistance in HIPEC for peritoneal metastases. These inhibitors target HSP90, reduce ATP levels, and induce pyroptosis, potentially converting immunologically “cold” tumors into “hot” ones and stimulating an antitumor immune response [169]. **Radiation Therapy**: Multi‐omic analysis in xenograft models revealed that combining radiation with an HSP90 inhibitor induces rapid adaptive changes in gene expression and pathway activity, suggesting potential targets for therapeutic intervention [182].

Clearly, the novel HSP90‐targeted drug modalities, advanced delivery systems, and rationally designed combination regimens are under active investigation to address these limitations. In parallel, the field has strategically pivoted to exploring combination therapies that leverage synergistic actions and target specific resistance pathways to improve outcomes.

## 7 ERS and Chaperone Mistranslocation

Moving beyond the traditional understanding of HSP90 and associated therapeutic strategies, recent studies have uncovered its stress‐induced mislocalization and spurred the development of novel rational approaches. GRP94 is the ER‐resident paralog of the HSP90 family and is strongly upregulated under stress conditions [183]. ER is a key membranous organelle in eukaryotic cells, responsible for protein synthesis and maturation, post‐translational processing, lipid biosynthesis, and calcium homeostasis [184]. Within the ER lumen, the buildup of aberrant proteins activates the unfolded protein response (UPR), a signaling cascade mediated by three major transmembrane sensors: inositol‐requiring enzyme 1 (IRE1), protein kinase R‐like ER kinase (PERK), and activating transcription factor 6 (ATF6). Under homeostatic conditions, these ER stress sensors are bound by luminal chaperones such as GRP78 and GRP94, which keep them in an inactive state [185]. When misfolded proteins accumulate, GRP78/GRP94 are titrated away from the sensors, leading to their activation. The UPR then orchestrates a multifaceted response that includes transient attenuation of global protein translation, upregulation of ER chaperones and folding enzymes, expansion of ER capacity, and, in cases of unresolved stress, engagement of pro‐apoptotic pathways. In cancer, chronic low‐grade UPR activation can support tumor cell survival, adaptation to a hostile microenvironment, and resistance to chemotherapy and targeted agents; conversely, excessive disruption of this proteostasis network can sensitize tumor cells and enhance therapeutic efficacy.

As the only HSP90 isoform localized within the ER lumen, GRP94 plays a critical role in ER‐associated protein folding and quality control. It selectively interacts with a defined subset of client proteins‐including Toll‐like receptors [186], the Wnt co‐receptor LRP6 [187], and GARP [188]‐ many of which are central to immune modulation and cancer progression. GRP94 is expressed across diverse cell types and is induced by interferons, as well as by conditions that compromise ER function, such as glucose deprivation, oxidative stress, depletion of ER calcium stores, and accumulation of misfolded proteins [189]. Notably, under certain circumstances, GRP94 can be found at the cell surface or in the extracellular space [190, 191]. Although the precise molecular routes by which ER chaperones‐including HSP90 family members‐reach the plasma membrane are not fully understood, several mechanistic models and partially validated pathways have been proposed [192].

The ER is also a central hub for protein trafficking. To maintain proper compartmentalization, ER‐resident chaperones must be distinguished from secretory cargo to prevent inadvertent secretion. This selectivity is mediated by C‐terminal ER‐retention motifs, such as Lys‐Asp‐Glu‐Leu (KDEL), found on proteins including BiP, GRP94, and protein disulfide isomerase (PDI) [193]. KDEL sequences are recognized by KDEL receptors (KDELRs), which cycle between the ER, the ER‐Golgi intermediate compartment (ERGIC), and the Golgi apparatus. KDELRs bind escaped KDEL‐bearing proteins in the Golgi and package them into COPI‐coated vesicles for retrograde transport, thereby ensuring efficient retrieval of these chaperones to the ER [194]. One long‐standing explanation for the presence of abundant ER proteins such as GRP94 on the cell surface is the “spillover” model. In this scenario, saturation or functional impairment of the KDELR‐mediated retrieval system allows a fraction of KDEL‐containing proteins to escape capture, proceed along the anterograde secretory pathway, and ultimately appear on the plasma membrane or be secreted. This concept was first proposed for GRP94 in 1987 and has since been extended to other KDEL‐containing proteins, including PDI and BiP [195].

Beyond passive spillover, GRP94 can form complexes that may facilitate its trafficking. It has been reported to associate with major histocompatibility complex class I (MHC‐I) molecules and immunoglobulin chains [196]. At the plasma membrane, GRP94 has been linked to oncogenic signaling; in HER2‐positive breast cancer, membrane‐associated GRP94 directly interacts with HER2 [115], promoting HER2 dimerization, phosphorylation, and activation of downstream signaling pathways, thereby contributing to tumor progression [116]. In castration‐resistant prostate cancer, cells under stress (e.g., heat shock) release extracellular vesicles enriched in chaperones and other stress‐induced components‐the so‐called “stressome”‐which may include GRP94 and related chaperones, providing an additional route for their extracellular accumulation [192].

For many cytosolic chaperones, surface localization appears to involve non‐canonical, non‐ERGIC/Golgi export pathways, potentially including direct translocation across the plasma membrane, association with exosomes or other vesicular carriers, or interactions with lumen‐facing phospholipid structures [197]. Importantly, ERS can alter the subcellular distribution of ER‐resident chaperones, promoting their re‐localization from the ER lumen to the plasma membrane and extracellular space [198]. This stress‐induced redistribution substantially broadens their functional repertoire, enabling roles in cell survival, migration, angiogenesis, and immune recognition.

Although the trafficking mechanisms are not fully defined, several signaling links between ER stress and chaperone mislocalization have been uncovered. For example, ERS can activate SRC family kinases, which in turn drive the dispersion and altered trafficking of KDELRs from the Golgi apparatus [199]. Partial loss of KDELR‐mediated retrograde transport facilitates the escape of KDEL‐bearing chaperones, including GRP94 and GRP78, to the cell surface. In AML, GRP94 and P4HB have been specifically detected on the surface of leukemic blasts, but not on normal hematopoietic cells. Treatment with the FLT3 inhibitor quizartinib markedly reduces their surface expression, suggesting that FLT3‐ITD driven signaling contributes to both ERS and chaperone translocation [191]. These observations have been exploited therapeutically: CAR immune cells engineered to target surface‐expressed chaperones show promising antitumor activity in preclinical AML models.

Antibody‐based targeting of cell surface GRP94 has also yielded encouraging results. Monoclonal antibodies directed against GRP94 can suppress tumor growth through multiple mechanisms [200], including inhibition of cetuximab‐resistant colorectal cancer cell proliferation and suppression of tumor angiogenesis, without causing severe systemic toxicity [201]. Because surface GRP94 and GRP78 tend to be enriched on stressed or transformed cells and are minimal on most normal tissues, they represent attractive tumor‐selective antigens. Thus, chaperone mislocalization, originally viewed as a by‐product of ER overload, is increasingly recognized as a functional phenotype that shapes tumor‐immune interactions and drug responses.

**FIGURE 6:**
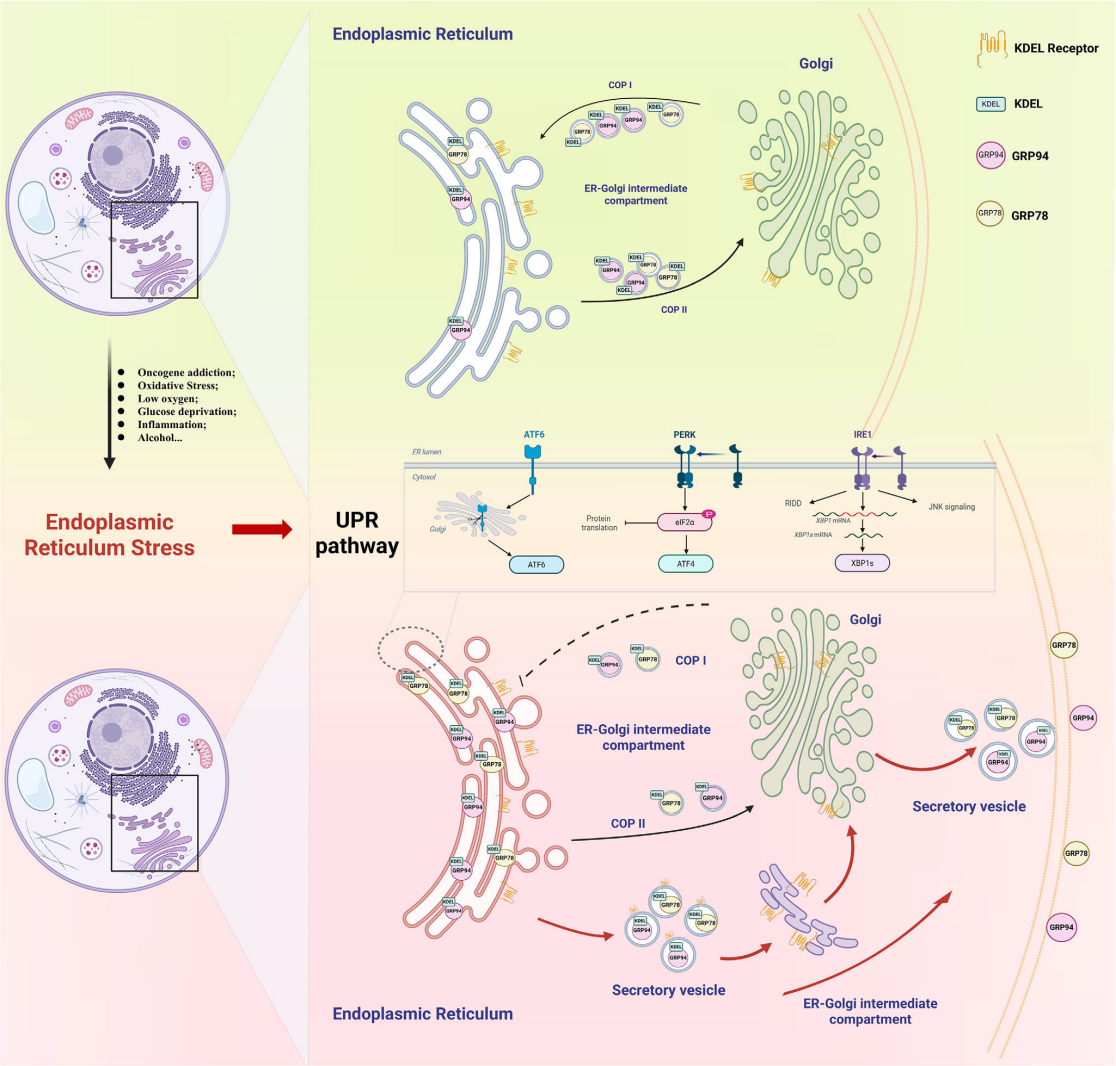
ER stress‐induced UPR mediates the relocalization of ER‐resident chaperone proteins.

Based on these observations, Figure 6 schematically illustrates potential mechanisms underlying the relocalization of ER‐resident proteins such as GRP94, closely linked to the UPR and ER stress. While similar pathways have been described for GRP78, GRP94 trafficking likely shares overlapping yet distinct routes, reflecting its specific client repertoire and interaction partners [202]. A deeper mechanistic understanding of ERS‐induced chaperone mislocalization may not only clarify how proteostasis stress rewires cell surface proteomes but also reveal new therapeutic entry points for antibody, chimeric antigen receptor (CAR), or ligand‐based strategies directed against tumor‐associated chaperones. The surface relocalization of chaperones under ER stress directly links proteostasis perturbations to immune recognition, prompting a closer examination of HSP90's broader roles in immune regulation and cancer immunotherapy.

## 8 Role of HSP90 in Immune Regulation and Immunotherapy

HSP90 modulates antigen presentation by antigen‐presenting cells (APCs), which display antigens on MHC molecules to potentially activate T cells, a process crucial for adaptive immunity, especially in cancer contexts. Notably, inhibition of HSP90 can increase the surface abundance of immunologically relevant receptors in malignant cells, including MHC‐I and the IFN‐gamma receptor (IFNGR) [203]. Under HSP90 inhibition, expression of immune‐related receptors on the surface of tumor cells is up‐regulated, which seemed to contradict the overall effect of HSP90 inhibition on enhancing anti‐tumor immunity. However, further analysis showed that there was an uncoupling between the change of receptor expression level and functional antigen presentation, which was rooted in the fine dependence of MHC‐I molecular antigen presentation on the chaperone network [204]. The normal function of the peptide loading complex of MHC‐I molecules is highly dependent on the involvement of HSP90 and its synergistic partners [205]. HSP90 inhibition can interfere with the assembly and stability of this complex, resulting in MHC‐I molecules being synthesized and transported to the cell membrane surface, but forming peptide‐deficient or unstable MHC‐I complexes due to the lack of stable loading of high‐affinity antigen peptides, thereby weakening their ability to effectively activate T cells [206]. Therefore, the upregulation of immune‐related receptor expression is not equivalent to the enhancement of antigen presentation function, and the asynchronous changes of the two under HSP90 inhibition reveal the complex dual role of molecular chaperones in regulating tumor immunogenicity [207]. On one hand, HSP90 inhibition enhances the “visibility” of tumor cells to immune effector cells by upregulating immune recognition receptors; on the other hand, to some extent, its interference with the antigenic peptide loading process might partially weaken the efficiency of MHC‐I restriction antigen presentation. This effect was consistently manifested across various cancer cell types with both cytosol HSP90‐targeted agents and pan‐HSP90 inhibitors, revealing a conserved and complex mechanism with broad applicability. Elevated IFNGR expression enhances tumor cell sensitivity to IFN‐γ, a cytokine from activated T and natural killer cells that mediates antitumor immunity. Thus, HSP90 inhibition may strengthen antigen presentation and IFN‐γ responsiveness, revealing a direct link between HSP90 activity and antitumor immunity. This supports the rationale for combining HSP90 inhibitors with immune checkpoint inhibitors (ICIs) to improve therapeutic outcomes [23, 203]. By converting immunologically “cold” tumors characterized by poor immune infiltration into “hot” immunogenic environments, HSP90 inhibitors may broaden the applicability and efficacy of cancer immunotherapies.

Immune checkpoints are vital for tolerance and balance, but tumors exploit them to evade immune detection, often by enhancing PD‐L1. HSP90 is key to this resistance strategy. The previous research demonstrates that HSP90 inhibitors can suppress IFN‐γ induced upregulation of immune checkpoints like IDO1 and PD‐L1 in cancer cell lines and primary cells [208]. Further evidence implicates HSP90 in regulating PD‐L1 via protein interactions: for example, PER2 binds to HSP90 and attenuates the interaction between IKKα/β and p65, this PER2‐HSP90 axis influences PD‐L1 expression and modulates anti‐tumor immunity. Combining PER2 targeting with anti PD‐L1 therapy has demonstrated the ability to enhance growth inhibition and CD8+ T‐cell infiltration in oral squamous cell carcinoma models, validating the potential of targeting HSP90‐related pathways to improve immune checkpoint blockade efficacy [209].

Isoform‐selective HSP90 inhibitors‐particularly those targeting Hsp90β‐offer a promising strategy for modulating immunoregulatory pathways while minimizing adverse effects associated with pan‐inhibitors. For instance, inhibition of Hsp90β upregulates IFN‐responsive genes and potentiates ICI activity in preclinical murine models. The Hsp90β inhibitor NDNB1182 downregulates CDK4 and induces IFN‐stimulated genes, leading to improved therapeutic responses and better tolerability relative to pan‐inhibitors such as ganetespib in models of PCa and BC [210]. HSP90 inhibition also directly affects regulatory T cells (Tregs), which are known to suppress immune responses and tend to be abundant within the TME, contributing to immunotherapy resistance [211]. In a Phase Ib trial of the oral HSP90 inhibitor TAS‐116 (pimitespib, the first approved HSP90 inhibitor drug) combined with nivolumab (anti PD‐1 antibody), biomarker analyses indicated that TAS‐116 suppressed Treg activity [212].

**FIGURE 7:**
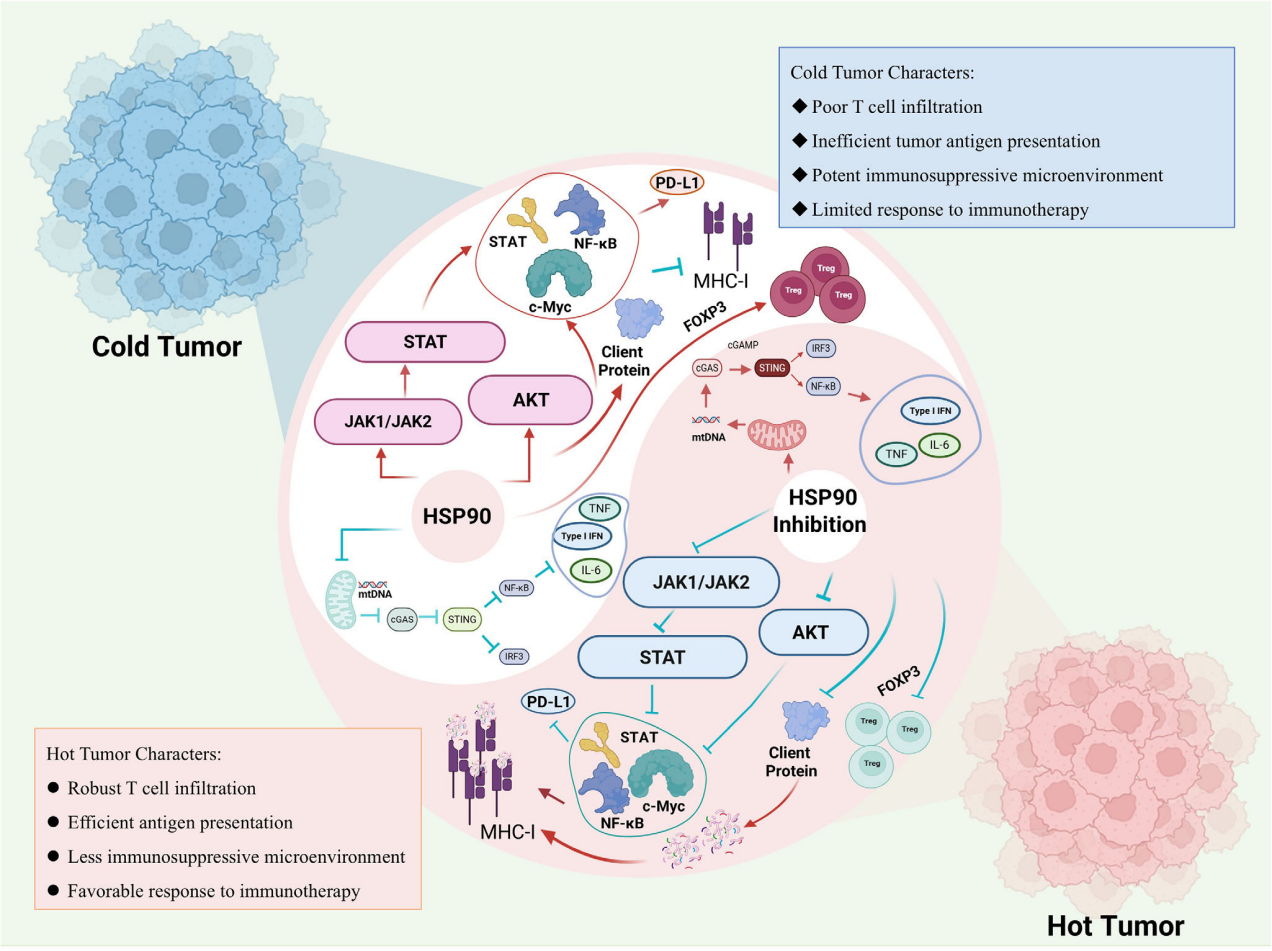
Potential roles of HSP90 in immune regulation and immunotherapy in malignancy.

The role of HSP90 in immune regulation is multifaceted. It modulates anti‐tumor immunity by regulating key factors within the TME, including antigen presentation, signaling pathways, immune checkpoint expression, and T‐cell function, thereby influencing whether a tumor is perceived as immunologically “cold” or “hot”. A summary of these mechanisms is provided in Figure 7. These functions establish HSP90 as a central mediator of cellular protein homeostasis and immune regulation, making it an attractive target for enhancing anti‐tumor immunity. Previous studies show that HSP90 inhibitors downregulate both protein and mRNA levels of PD‐L1 and PD‐L2, potentially through transcriptional regulators such as STAT‐1 and c‐Myc [213]. In preclinical models, HSP90 inhibition suppresses tumor‐cell PD‐L1 expression while enhancing CD8+ T‐cell cytotoxicity and infiltration, suggesting a dual mechanism of action [214]. Specific inhibitors like ganetespib (STA‐9090) hold therapeutic potential for combination immunotherapy, as they enhance anti‐tumor immunity by suppressing PD‐L1 [215]. Additionally, HSP90‐targeting agents can induce mitochondrial stress and intrinsic apoptosis, activating innate immune responses through the mitochondrial DNA‐cGAS‐STING‐IRF3 axis [216]. Moreover, inhibiting of Caspase9 triggers HSP90‐based chemotherapy, amplifies type I IFN production and, in combination with anti‐PD‐L1 therapy, promotes tumor regression in murine models [217].

HSP90 inhibition has been shown to upregulate pro‐inflammatory cytokines (e.g., IL‐6, IL‐1β) and chemokines (e.g., CCL2, CXCL1) in various cell types [218, 219], factors known to mediate MDSC recruitment and function [220, 221]. While direct evidence linking HSP90 inhibition to increased MDSC infiltration remains limited, the activation of STAT3 [222], NF‐κB [209], and HIF‐1α pathway suggests a potential for context‐dependent immunomodulatory effects. Notably, studies have also demonstrated that HSP90 inhibition can reduce MDSC infiltration when combined with immune checkpoint blockade [223, 224], highlighting the complexity of its impact on the TME. Further investigation is warranted to determine whether compensatory MDSC accumulation occurs under specific dosing or temporal conditions. Moreover, T‐cell exhaustion (T_e__x_), a hypofunctional state marked by impaired effector activity and elevated expression of inhibitory receptors like PD‐1 and TIM3, represents a major contributor to immunotherapy resistance. Recent research reveals that exhausted CD8+ T cells exhibit a unique proteotoxic stress response (T_e__x_‐PSR), marked by enhanced global translation, upregulation of chaperones (e.g., GRP94/GP96, GRP78/BiP), and accumulation of protein aggregates [225]. This response is driven by sustained AKT signaling and directly promotes the transition from effector T cells to T_e__x_ cells. Disruption of T_e__x_‐PSR‐associated chaperones improves immunotherapy efficacy in preclinical models, and high T_e__x_‐PSR in patient T cells correlates with poor treatment response, indicating that T_e__x_‐PSR serves as both a marker and a driver of T‐cell exhaustion, offering new therapeutic targets. Chimeric antigen receptor (CAR) immune cells, such as CAR‐T and CAR‐NK cells, have emerged as a transformative modality in cancer immunotherapy [226]. Preclinical evaluations of GRP78‐specific CAR‐T cells have demonstrated potent cytotoxic activity against diverse malignancies, including AML [227], GBM [228], lung cancer [229], highlighting its potential as a pan‐cancer immunotherapy target. Notably, GRP78 and GRP94‐key regulators of ERS‐are often co‐expressed in the TME, highlighting the potential of chaperone‐targeting CAR strategies. For example, CAR‐NK cells targeting cell surface GRP94 show augmented expression of CD107a, granzyme B, as well as IFN‐γ when co‐cultured with the cell surface GRP94‐positive malignant cells, showing specific cytotoxicity against cancer cells without affecting healthy cells [191].

Given the key role of HSP90 in maintaining signaling and functional homeostasis, HSP90 inhibitors may have multiple effects on effector immune cells while exerting anti‐tumor effects. Tumor cells are in a state of continuous stress, showing “non‐oncogene addiction” to HSP90, and their protein homeostasis is highly dependent on the maintenance of HSP90 [13, 14]. HSP90 is frequently overexpressed in many tumor types and is closely associated with tumor malignancy and poor prognosis. Therefore, the protein homeostasis of tumor cells might be highly dependent on HSP90, which means that HSP90 inhibitors could induce tumor cell apoptosis at lower doses or with shorter exposure durations. In contrast, although T cells and NK cells similarly depend on HSP90 for functional maintenance, they might endure the HSP90 inhibition rather than cell death under equivalent conditions. Some studies have indicated that the HSP90 inhibitor pimitespib can reduce the highly immunosuppressive FOXP3 effector Treg cells, which improves the priming and activation of the antigen‐specific CD8+ T cells [213]. Moreover, HSP90 inhibition enhances cancer immunotherapy by modulating the surface expression of multiple immune checkpoint proteins [230]. This different sensitivity provides a therapeutic window for HSP90 inhibitors between tumors and immune cells, which provides a theoretical basis for the HSP90 targeted therapy.

## 9 Conclusion

**FIGURE 8:**
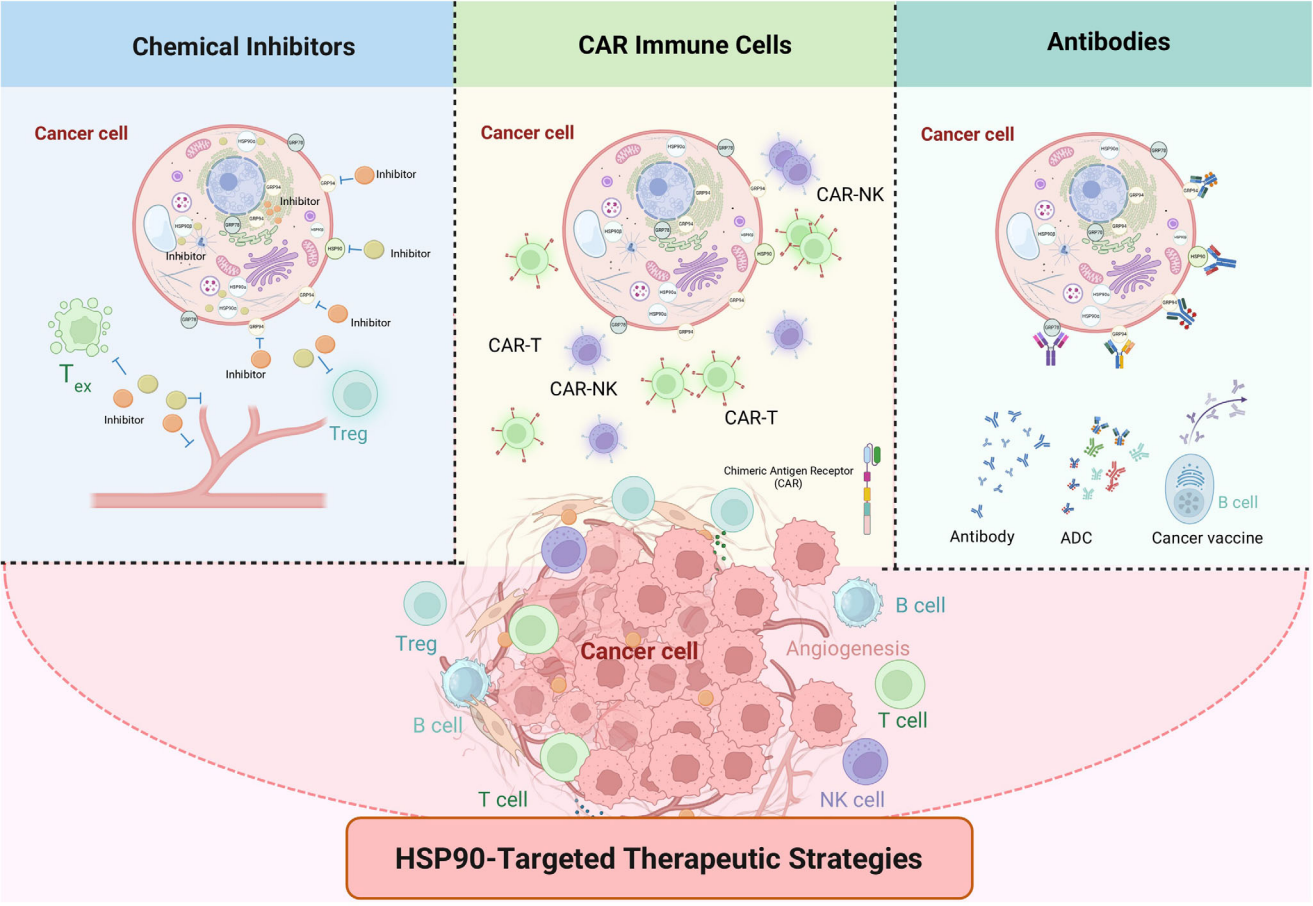
The current landscape of HSP90 targeted therapeutic strategies in cancer therapy.

HSP90 is an indispensable molecular chaperone that regulates the folding, stability, and activity of a vast array of client proteins vital for maintaining cellular homeostasis. Its profound involvement in maintaining proteostasis, coupled with frequent overexpression and altered activity in pathological states‐particularly cancer‐has established it as a highly attractive therapeutic target. HSP90's chaperone cycle and co‐chaperone interactions underpin its cellular functions. In cancer, HSP90 stabilizes oncoproteins to drive tumor growth, survival, and drug resistance. HSP90 inhibition enhances antigen presentation via MHC‐I and IFNGR, and also reduces immunosuppression by destabilizing STATs and downregulating PD‐L1 and IDO1. HSP90 further shapes the tumor immune landscape by influencing immune cell function and polarization. HSP90 inhibitors show growing promise in immunotherapy. These agents synergize with immune checkpoint blockade, thermal therapy, radiotherapy, and chemotherapy by enhancing immune responses and overcoming resistance. When combined with nanomedicine, they enable targeted delivery and reduced systemic toxicity. This review has elucidated the diverse and multifaceted roles of HSP90, not only in driving oncogenesis but also in modulating drug resistance and immune responses, thereby revealing novel avenues for disease therapy. Taken together, HSP90 chemical inhibitors and several novel strategies like CAR technologies and antibody‐associated strategies represent significant advances in oncology targeted therapy (Figure 8). In addition, HSPs play a significant role in mediating therapy resistance to various anti‐cancer treatments [231]. Given the limited efficacy of HSP90 inhibitors as monoagents and the obvious heterogeneous response, future clinical translation needs to focus on biomarker‐based patient stratification strategies. As mentioned earlier, several studies have explored methods to predict response by detecting complex abundance in tumor tissue through PET imaging. In addition, the expression level of HSP90α/β in tumor tissue, HSF1 activation status, and the dependence of specific client proteins (e.g., HER2, EGFR) may also be used as markers for screening potential beneficiary populations. Combined with liquid biopsy to detect the expression of HSP90 and related chaperones in circulating tumor cells, or to analyze the activity of heat shock response pathways through tumor transcriptomics, it is expected to achieve more accurate patient stratification.

However, there remain unresolved controversies in this field, including how to develop reliable predictive biomarkers for patient stratification, how to comprehensively elucidate the complex non‐cell‐dependent mechanisms of HSP90 in the tumor immune microenvironment, and how to design more precise delivery systems to overcome toxicity while exploring novel intervention strategies targeting specific HSP90 subtypes or post‐translational modifications. The realistic feasibility of these strategies is being validated in prospective clinical trials and will open new avenues for personalized precision cancer treatment focusing on resistance and recurrence. Future studies should aim to elucidate the underlying molecular mechanisms, optimize therapeutic regimens, and improve patient outcomes.

## Author Contributions

**Beibei Zhang**, **Xinxin Zou**, and **Jiansong Zhou** contributed equally to this work. **Xinxin Zou**: writing – original draft, visualization. **Dahua Chen**: conceptualization, writing – original draft, writing – review and editing, visualization, funding acquisition, project administration, and supervision. **Beibei Zhang**: conceptualization, writing – original draft, writing – review and editing, project administration, funding acquisition, visualization, and supervision. **Jiansong Zhou**: writing – original draft, and visualization. **Qinmiao Sun**: conceptualization, writing – review and editing, visualization.

## Funding

This research was supported by Excellence Research Group Program of NSFC (32588201), National Natural Science Foundation of China (Grant no. 82260038), Yunnan Province Science and Technology Department (Grant no. 202305AT350003, 202305AS350022, 202105AB160002, 202601AS070039, and 202501AT070207), and Basic Science Center Program of NSFC (Grant no. 31988101).

## Consent

The authors declare that no patient or participant consent was required for this study.

## Declaration of Generative AI Use

The authors declare that no Generative AI was used in the creation of this manuscript.

## Conflicts of Interest

The authors declare no competing interests.

## Data Availability

Data sharing not applicable to this article as no datasets were generated or analyzed during the current study.

## References

[1] G. I. Karras , G. Colombo , and A. N. Kravats , “Hsp30: Hsp90: Bringing it All Together,” Cell Stress and Chaperones 30, no. 1 (2025): 69–79, 10.1016/j.cstres.2025.01.002. 39889818 PMC12013134

[2] T. Morán Luengo , M. P. Mayer , and S. G. D. Rüdiger , “The Hsp70–Hsp90 Chaperone Cascade in Protein Folding,” Trends in Cell Biology 29, no. 2 (2019): 164–177, 10.1016/j.tcb.2018.10.004. 30502916

[3] T. Lacey and H. Lacey , “Linking hsp90's Role as an Evolutionary Capacitator to the Development of Cancer,” Cancer Treatment and Research Communications 28 (2021): 100400. 34023771 10.1016/j.ctarc.2021.100400

[4] P. Somu , S. Mohanty , N. Basavegowda , A. K. Yadav , S. Paul , and K.‐H. Baek , “The Interplay Between Heat Shock Proteins and Cancer Pathogenesis: A Novel Strategy for Cancer Therapeutics,” Cancers 16, no. 3 (2024): 638, 10.3390/cancers16030638. 38339390 PMC10854888

[5] L. Xiao , P. Rasouli , and D. Ruden , “Possible Effects of Early Treatments of hsp90 Inhibitors on Preventing the Evolution of Drug Resistance to Other Anti‐Cancer Drugs,” Current Medicinal Chemistry 14, no. 2 (2007): 223–232, 10.2174/092986707779313372. 17266581

[6] K. Jhaveri and S. Modi , “HSP90 Inhibitors for Cancer Therapy and Overcoming Drug Resistance,” Advances in Pharmacology 65 (2012): 471–517. 22959035 10.1016/B978-0-12-397927-8.00015-4

[7] A. Singh , S. Maity , P. Devi , A. Rai , and V. Asati , “Recent Progress and Structural Insights of Potential Hsp90 Inhibitors as Anticancer Agents,” Molecular Diversity 29, no. 6 (2025): 5335–5366, 10.1007/s11030-025-11160-3. 40100483

[8] X. Liang , R. Chen , C. Wang , Y. Wang , and J. Zhang , “Targeting HSP90 for Cancer Therapy: Current Progress and Emerging Prospects,” Journal of Medicinal Chemistry 67, no. 18 (2024): 15968–15995, 10.1021/acs.jmedchem.4c00966. 39256986

[9] L. Whitesell and S. L. Lindquist , “HSP90 and the Chaperoning of Cancer,” Nature Reviews Cancer 5, no. 10 (2005): 761–772, 10.1038/nrc1716. 16175177

[10] M. Wang , A. Shen , C. Zhang , et al., “Development of Heat Shock Protein (Hsp90) Inhibitors To Combat Resistance to Tyrosine Kinase Inhibitors Through Hsp90–Kinase Interactions,” Journal of Medicinal Chemistry 59, no. 12 (2016): 5563–5586, 10.1021/acs.jmedchem.5b01106. 26844689

[11] M. R. Amoroso , D. S. Matassa , I. Agliarulo , et al., “Stress‐Adaptive Response in Ovarian Cancer Drug Resistance: Role of TRAP1 in Oxidative Metabolism‐Driven Inflammation,” Adv Protein Chem Struct Biol 108 (2017): 163–198. 28427560 10.1016/bs.apcsb.2017.01.004

[12] X. Lu , L. Xiao , L. Wang , and D. M. Ruden , “Hsp90 Inhibitors and Drug Resistance in Cancer: The Potential Benefits of Combination Therapies of Hsp90 Inhibitors and Other Anti‐Cancer Drugs,” Biochemical Pharmacology 83, no. 8 (2012): 995–1004, 10.1016/j.bcp.2011.11.011. 22120678 PMC3299878

[13] P. Kumar , B. Devaki , U. K. Jonnala , and S. Amere Subbarao , “Hsp90 facilitates Acquired Drug Resistance of Tumor Cells Through Cholesterol Modulation However Independent of Tumor Progression,” Biochimica et Biophysica Acta (BBA)—Molecular Cell Research 1867, no. 8 (2020): 118728, 10.1016/j.bbamcr.2020.118728. 32343987

[14] N. A. Bacon , I. Larre , A. A. Lawag , et al., “Low Dose HSP90 Inhibition With AUY922 Blunts Rapid Evolution of Metastatic and Drug Resistant Phenotypes Induced by TGF‐β and Paclitaxel in A549 Cells,” Biomedicine & Pharmacotherapy 129 (2020): 110434, 10.1016/j.biopha.2020.110434. 32768937 PMC7484166

[15] O. Abdullah and Z. Omran , “Geldanamycins: Potent Hsp90 Inhibitors With Significant Potential in Cancer Therapy,” International Journal of Molecular Sciences 25, no. 20 (2024): 11293, 10.3390/ijms252011293. 39457075 PMC11509085

[16] T. Isermann , K. L. Schneider , F. Wegwitz , et al., “Enhancement of Colorectal Cancer Therapy Through Interruption of the HSF1‐HSP90 Axis by p53 Activation or Cell Cycle Inhibition,” Cell Death & Differentiation 32, no. 9 (2025): 1734–1749, 10.1038/s41418-025-01502-x. 40204953 PMC12432187

[17] Z. Wu , Y. Geng , X. Lu , et al., “Chaperone‐Mediated Autophagy Is Involved in the Execution of Ferroptosis,” Proceedings of the National Academy of Sciences 116, no. 8 (2019): 2996–3005, 10.1073/pnas.1819728116. PMC6386716 30718432

[18] S. Mallick , V. K. Narayana , A. B. Rai , et al., “Hematopoietic Stem Cell Conditioned Media Induces Apoptosis in Colorectal Cancer Stem Cells via Dysregulation of HSP90 and 26S Proteasome System,” The International Journal of Biochemistry & Cell Biology 184 (2025): 106773, 10.1016/j.biocel.2025.106773. 40210089

[19] G. Lettini , S. Lepore , F. Crispo , L. Sisinni , F. Esposito , and M. Landriscina , “Heat Shock Proteins in Cancer Stem Cell Maintenance: A Potential Therapeutic Target?,” Histology and Histopathology 35, no. 1 (2020): 25–37. 31322279 10.14670/HH-18-153

[20] K. Ziaka and J. van der Spuy , “The Role of Hsp90 in Retinal Proteostasis and Disease,” Biomolecules 12, no. 7 (2022): 978, 10.3390/biom12070978. 35883534 PMC9313453

[21] Y. Zhang , X. Chen , B. Hu , B. Zou , and Y. Xu , “Advancements in Nanomedicine Delivery Systems: Unraveling Immune Regulation Strategies for Tumor Immunotherapy,” Nanomedicine 19, no. 21‐22 (2024): 1821–1840. 39011582 10.1080/17435889.2024.2374230 PMC11418288

[22] Y. Kim , S. Y. Lim , H. O. Kim , et al., “Combination Strategies with HSP90 Inhibitors in Cancer Therapy: Mechanisms, Challenges, and Future Perspectives,” Pharmaceuticals (Basel) 18, no. 8 (2025): 1083. 40872476 10.3390/ph18081083 PMC12389308

[23] S. Jia , N. Maurya , B. S. J. Blagg , and X. Lu , “Hsp90 pan and Isoform‐Selective Inhibitors as Sensitizers for Cancer Immunotherapy,” Pharmaceuticals 18, no. 7 (2025): 1025, 10.3390/ph18071025. 40732313 PMC12299041

[24] C. Zhang , W. Nie , Q. Tu , Y. Zhang , H. Zhao , and C. Song , “HSP90 inhibitors in Cancer Immunotherapy: Therapeutic Opportunities and Challenges,” European Journal of Medicinal Chemistry 301 (2026): 118195, 10.1016/j.ejmech.2025.118195. 41037985

[25] R. Kumar R , N. N S , A. S P , et al., “HSPIR: A Manually Annotated Heat Shock Protein Information Resource,” Bioinformatics 28, no. 21 (2012): 2853–2855. 22923302 10.1093/bioinformatics/bts520 PMC3476333

[26] V. A. Lopez , B. C. Park , D. Nowak , et al., “A Bacterial Effector Mimics a Host HSP90 Client to Undermine Immunity,” Cell 179, no. 1 (2019): 205–218.e21, 10.1016/j.cell.2019.08.020. 31522888 PMC6754304

[27] J. Zhang , H. Li , Y. Liu , et al., “Targeting HSP90 as a Novel Therapy for Cancer: Mechanistic Insights and Translational Relevance,” Cells 11, no. 18 (2022): 2778. 36139353 10.3390/cells11182778 PMC9497295

[28] C. Prodromou , “Mechanisms of Hsp90 Regulation,” Biochemical Journal 473, no. 16 (2016): 2439–2452, 10.1042/BCJ20160005. 27515256 PMC4980810

[29] D. Picard , “Heat‐shock Protein 90, a Chaperone for Folding and Regulation,” Cellular and Molecular Life Sciences 59, no. 10 (2002): 1640–1648, 10.1007/PL00012491. 12475174 PMC11337538

[30] B. T. Lai , N. W. Chin , A. E. Stanek , W. Keh , and K. W. Lanks , “Quantitation and Intracellular Localization of the 85K Heat Shock Protein by Using Monoclonal and Polyclonal Antibodies,” Molecular and Cellular Biology 4, no. 12 (1984): 2802–2810. 6396506 10.1128/mcb.4.12.2802-2810.1984 PMC369291

[31] F. W. Farrelly and D. B. Finkelstein , “Complete Sequence of the Heat Shock‐inducible HSP90 Gene of Saccharomyces Cerevisiae,” Journal of Biological Chemistry 259, no. 9 (1984): 5745–5751, 10.1016/S0021-9258(18)91077-X. 6325446

[32] K. Vermeulen , E. Naus , M. Ahamed , et al., “Evaluation of [ 11 C]NMS‐E973 as a PET Tracer for In Vivo Visualisation of HSP90,” Theranostics 9, no. 2 (2019): 554–572, 10.7150/thno.27213. 30809293 PMC6376183

[33] M. Reidy and D. C. Masison , “Mutations in the Hsp90 N Domain Identify a Site That Controls Dimer Opening and Expand Human Hsp90α Function in Yeast,” Journal of Molecular Biology 432, no. 16 (2020): 4673–4689, 10.1016/j.jmb.2020.06.015. 32565117 PMC7437358

[34] Y. Zhang , F.‐M. Chang , J. Huang , et al., “DSSylation, a Novel Protein Modification Targets Proteins Induced by Oxidative Stress, and Facilitates Their Degradation in Cells,” Protein & Cell 5, no. 2 (2014): 124–140, 10.1007/s13238-013-0018-8. 24515614 PMC3956975

[35] J. L. M. Kotler and T. O. Street , “Mechanisms of Protein Quality Control in the Endoplasmic Reticulum by a Coordinated Hsp40‐Hsp70‐Hsp90 System,” Annual Review of Biophysics 52 (2023): 509–524, 10.1146/annurev-biophys-111622-091309. 37159299

[36] M. K. Singh , Y. Shin , S. Han , et al., “Molecular Chaperonin HSP60: Current Understanding and Future Prospects,” International Journal of Molecular Sciences 25, no. 10 (2024): 5483, 10.3390/ijms25105483. 38791521 PMC11121636

[37] N. B. Gusev , O. V. Bukach , and S. B. Marston , “Structure, Properties, and Probable Physiological Role of Small Heat Shock Protein With Molecular Mass 20 kD (Hsp20, HspB6),” Biochemistry (Moscow) 70, no. 6 (2005): 629–637, 10.1007/s10541-005-0162-8. 16038604

[38] S. Kedzierska‐Mieszkowska and M. Zolkiewski , “Hsp100 Molecular Chaperone ClpB and Its Role in Virulence of Bacterial Pathogens,” International Journal of Molecular Sciences 22, no. 10 (2021): 5319, 10.3390/ijms22105319. 34070174 PMC8158500

[39] G. E. Karagöz and S. G. D. Rüdiger , “Hsp90 interaction With Clients,” Trends in Biochemical Sciences 40, no. 2 (2015): 117–125. 25579468 10.1016/j.tibs.2014.12.002

[40] C. Prodromou , B. Panaretou , S. Chohan , et al., “The ATPase Cycle of Hsp90 Drives a Molecular ‘Clamp’ via Transient Dimerization of the N‐Terminal Domains,” The EMBO Journal 19, no. 16 (2000): 4383–4392, 10.1093/emboj/19.16.4383. 10944121 PMC302038

[41] C. K. Vaughan , P. W. Piper , L. H. Pearl , and C. Prodromou , “A Common Conformationally Coupled ATPase Mechanism for Yeast and Human Cytoplasmic HSP90s,” The FEBS Journal 276, no. 1 (2009): 199–209, 10.1111/j.1742-4658.2008.06773.x. 19032597 PMC2702006

[42] V. Narayanankutty , A. Narayanankutty , and A. Nair , “Heat Shock Proteins (HSPs): A Novel Target for Cancer Metastasis Prevention,” Current Drug Targets 20, no. 7 (2019): 727–737, 10.2174/1389450120666181211111815. 30526455

[43] I. Aolymat , M. M. Hatmal , and A. N. Olaimat , “The Emerging Role of Heat Shock Factor 1 (HSF1) and Heat Shock Proteins (HSPs) in Ferroptosis,” Pathophysiology 30, no. 1 (2023): 63–82, 10.3390/pathophysiology30010007. 36976734 PMC10057451

[44] S. Pawaria and R. Binder , “Role of CD91 in HSP‐Mediated Innate Immunity (165.9),” The Journal of Immunology 186, no. 1 (2011): 165.169–165.169.

[45] A. K. Ho , F. Jeganathan , M. Bictash , and H.‐J. Chen , “Identification of Novel Small Molecule Chaperone Activators for Neurodegenerative Disease Treatment,” Biomedicine & Pharmacotherapy 187 (2025): 118049, 10.1016/j.biopha.2025.118049. 40239269 PMC12086176

[46] S. D. Westerheide , R. Raynes , C. Powell , B. Xue , and V. N. Uversky , “HSF Transcription Factor family, Heat Shock Response, and Protein Intrinsic Disorder,” Current Protein & Peptide Science 13, no. 1 (2012): 86–103, 10.2174/138920312799277956. 22044151

[47] S. Dayalan Naidu and A. T. Dinkova‐Kostova , “Regulation of the Mammalian Heat Shock Factor 1,” The FEBS Journal 284, no. 11 (2017): 1606–1627, 10.1111/febs.13999. 28052564

[48] Y. Shi , D. D. Mosser , and R. I. Morimoto , “Molecularchaperones as HSF1‐Specific Transcriptional Repressors,” Genes & Development 12, no. 5 (1998): 654–666, 10.1101/gad.12.5.654. 9499401 PMC316571

[49] M. L. Mendillo , S. Santagata , M. Koeva , et al., “HSF1 Drives a Transcriptional Program Distinct From Heat Shock to Support Highly Malignant Human Cancers,” Cell 150, no. 3 (2012): 549–562, 10.1016/j.cell.2012.06.031. 22863008 PMC3438889

[50] C. Dai , “The Heat‐Shock, or HSF1‐Mediated Proteotoxic Stress, Response in Cancer: From Proteomic Stability to Oncogenesis,” Philosophical Transactions of the Royal Society B: Biological Sciences 373, no. 1738 (2018): 20160525, 10.1098/rstb.2016.0525. PMC5717525 29203710

[51] Z. Albakova , “HSP90 Multi‐Functionality in Cancer,” Frontiers in Immunology 15 (2024): 1436973, 10.3389/fimmu.2024.1436973. 39148727 PMC11324539

[52] Y. Zhang , G. Zhao , L. Yu , et al., “Heat‐Shock Protein 90α Protects NME1 Against Degradation and Suppresses Metastasis of Breast Cancer,” British Journal of Cancer 129, no. 10 (2023): 1679–1691, 10.1038/s41416-023-02435-3. 37731021 PMC10645775

[53] N. AbdElmoniem , M. H. Abdallah , R. M. Mukhtar , et al., “Identification of Novel Natural Dual HDAC and Hsp90 Inhibitors for Metastatic TNBC Using e‐Pharmacophore Modeling, Molecular Docking, and Molecular Dynamics Studies,” Molecules 28, no. 4 (2023): 1771. 36838758 10.3390/molecules28041771 PMC9965823

[54] J. Y. Kim , T.‐M. Cho , J. M. Park , et al., “A Novel HSP90 Inhibitor SL‐145 Suppresses Metastatic Triple‐Negative Breast Cancer Without Triggering the Heat Shock Response,” Oncogene 41, no. 23 (2022): 3289–3297, 10.1038/s41388-022-02269-y. 35501463 PMC9166677

[55] E. Jung , S. Jang , D. Sung , et al., “Abstract 4911: A Novel C‐Terminal of HSP90 Inhibitor NCT‐547 Eliminates Cancer Stem‐Like Subpopulation in Triple‐Negative Breast Cancer,” Cancer Research 83, no. 7 (2023): 4911–4911, 10.1158/1538-7445.AM2023-4911.

[56] I. Zarguan , S. Lu , vamsi Krishna Kommalapati , et al., “Abstract 3055: Developing the Cyclic Peptide AD05 as a Novel Hsp90 Inhibitor for Triple‐Negative Breast Cancer Therapy,” Cancer Research 85, no. 8 (2025): 3055–3055, 10.1158/1538-7445.AM2025-3055.

[57] C. P. Ogbu , T. Llbiyi , V. K. Kommalapati , and A. Chadli , “Abstract 85: Abstract 6780: Discovering Novel Inhibitors of the Chaperone Hsp90 for Cancer Treatment: A Plant‐Based Approach,” Cancer Research 85, no. 8 (2025): 6780–6780, 10.1158/1538-7445.AM2025-6780.

[58] Z. Zhang , F. Tian , S. Lai , et al., “Suppression of the HSP90‐HIF1α Pathway With SNX2112‐Encapsulated Nano‐Micelles for Effective Triple‐Negative Breast Cancer Photothermal Combined Photodynamic Therapy,” Journal of Materials Chemistry B 13, no. 26 (2025): 7753–7768, 10.1039/D5TB00071H. 40468772

[59] H. Kim , E. Elkins , R. Islam , et al., “Silmitasertib (CX‐4945) Disrupts ERα/HSP90 Interaction and Drives Proteolysis Through the Disruption of CK2β Function in Breast Cancer Cells,” Cancers 16, no. 14 (2024): 2501, 10.3390/cancers16142501. 39061141 PMC11274397

[60] L. Cis , M. G. Zamperla , V. Barbi , et al., “Abstract 2977: Novel HSP90 Inhibitors With Senolytic Activity for a Targeted Therapy in a Hormone‐Induced Breast Cancer Senescence Model,” Cancer Research 84, no. 6 (2024): 2977–2977, 10.1158/1538-7445.AM2024-2977.

[61] G. L. Monica , F. Alamia , A. Bono , et al., “In Silico Design of Dual Estrogen Receptor and Hsp90 Inhibitors for ER‐Positive Breast Cancer Through a Mixed Ligand/Structure‐Based Approach,” Molecules 29, no. 24 (2024): 6040, 10.3390/molecules29246040. 39770128 PMC11676166

[62] I. Falcone , E. Giontella , S. Giuliani , et al., “HER2‐Driven Breast Cancer: Role of the Chaperonin HSP90 in Modulating Response to Trastuzumab‐Based Therapeutic Combinations,” International Journal of Molecular Sciences 26, no. 14 (2025): 6593, 10.3390/ijms26146593. 40724844 PMC12294885

[63] J. M. Park , Y.‐J. Kim , S. Park , et al., “A Novel HSP90 Inhibitor Targeting the C‐Terminal Domain Attenuates Trastuzumab Resistance in HER2‐Positive Breast Cancer,” Molecular Cancer 19, no. 1 (2020): 161, 10.1186/s12943-020-01283-6. 33218356 PMC7678296

[64] K. Jhaveri , R. Wang , E. Teplinsky , et al., “A Phase I Trial of Ganetespib in Combination With Paclitaxel and Trastuzumab in Patients With human Epidermal Growth Factor Receptor‐2 (HER2)‐Positive Metastatic Breast Cancer,” Breast Cancer Research 19, no. 1 (2017): 89, 10.1186/s13058-017-0879-5. 28764748 PMC5540198

[65] S. Modi , C. Saura , C. Henderson , et al., “A Multicenter Trial Evaluating Retaspimycin HCL (IPI‐504) plus Trastuzumab in Patients With Advanced or Metastatic HER2‐positive Breast Cancer,” Breast Cancer Research and Treatment 139, no. 1 (2013): 107–113, 10.1007/s10549-013-2510-5. 23580070 PMC3646160

[66] C. E. Eyermann , J. D. Haley , E. M. Alexandrova , et al., “The HSP‐RTK‐Akt Axis Mediates Acquired Resistance to Ganetespib in HER2‐Positive Breast Cancer,” Cell Death & Disease 12, no. 1 (2021): 126, 10.1038/s41419-021-03414-3. 33500390 PMC7838268

[67] X. Gao , X. Guo , W. Yuan , et al., “Pyrotinib Induces Cell Death in HER2‐Positive Breast Cancer via Triggering HSP90‐Dependent HER2 Degradation and ROS/HSF‐1‐Dependent Oxidative DNA Damage,” Cell Stress and Chaperones 29, no. 6 (2024): 777–791, 10.1016/j.cstres.2024.11.004. 39566595 PMC11653144

[68] T. Shimamura and G. I. Shapiro , “Heat Shock Protein 90 Inhibition in Lung Cancer,” Journal of Thoracic Oncology 3, no. 6 (2008): S152–S159, 10.1097/JTO.0b013e318174ea3a. 18520302 PMC4475350

[69] C.‐H. Lai , K.‐S. Park , D.‐H. Lee , et al., “HSP‐90 Inhibitor Ganetespib Is Synergistic With Doxorubicin in Small Cell Lung Cancer,” Oncogene 33, no. 40 (2014): 4867–4876, 10.1038/onc.2013.439. 24166505 PMC4002667

[70] A. Truini , J. H. Starrett , T. Stewart , et al., “The EGFR Exon 19 Mutant L747‐A750>P Exhibits Distinct Sensitivity to Tyrosine Kinase Inhibitors in Lung Adenocarcinoma,” Clinical Cancer Research 25, no. 21 (2019): 6382–6391. 31182434 10.1158/1078-0432.CCR-19-0780 PMC6825535

[71] G. Multhoff and J. Radons , “Radiation, Inflammation, and Immune Responses in Cancer,” Frontiers in Oncology 2 (2012): 58, 10.3389/fonc.2012.00058. 22675673 PMC3366472

[72] Y. Sun , Y.‐H. Huang , F.‐Y. Huang , et al., “3′‐epi‐12β‐hydroxyfroside, a New Cardenolide, Induces Cytoprotective Autophagy via Blocking the Hsp90/Akt/mTOR Axis in Lung Cancer Cells,” Theranostics 8, no. 7 (2018): 2044–2060, 10.7150/thno.23304. 29556372 PMC5858516

[73] S. B. M. Schmitz , J. Gülden , M. Niederreiter , C. Eichner , J. Werner , and B. Mayer , “Strong Hsp90α/β Protein Expression in Advanced Primary CRC Indicates Short Survival and Predicts Response to the Hsp90α/β‐Specific Inhibitor Pimitespib,” Cells 14, no. 11 (2025): 836, 10.3390/cells14110836. 40498011 PMC12154481

[74] D. He , et al., “Icaritin Represses Autophagy to Promote Colorectal Cancer Cell Apoptosis and Sensitized Low‐Temperature Photothermal Therapy via Targeting HSP90‐TXNDC9 Interactions,” Advanced Science 12, no. 20 (2025): 2412953. 40184625 10.1002/advs.202412953 PMC12120733

[75] Z. Lin , R. Pan , L. Wu , et al., “AFP‐HSP90 Mediated MYC/MET Activation Promotes Tumor Progression in Hepatocellular Carcinoma and Gastric Cancers,” Cancer Cell International 24, no. 1 (2024): 283, 10.1186/s12935-024-03455-6. 39135041 PMC11321088

[76] G. Floris , R. Sciot , A. Wozniak , et al., “The Novel HSP90 Inhibitor, IPI‐493, Is Highly Effective in Human Gastrostrointestinal Stromal Tumor Xenografts Carrying Heterogeneous KIT Mutations,” Clinical Cancer Research 17, no. 17 (2011): 5604–5614, 10.1158/1078-0432.CCR-11-0562. 21737509

[77] S. Liu , G. Shen , X. Zhou , et al., “Hsp90 Promotes Gastric Cancer Cell Metastasis and Stemness by Regulating the Regional Distribution of Glycolysis‐Related Metabolic Enzymes in the Cytoplasm,” Advanced Science 11, no. 33 (2024): 2310109. 38874476 10.1002/advs.202310109 PMC11434123

[78] C. Duan , K. Li , X. Pan , Z. Wei , and L. Xiao , “Hsp90 is a Potential Risk Factor for Ovarian Cancer Prognosis: An Evidence of a Chinese Clinical Center,” BMC cancer 23, no. 1 (2023): 489, 10.1186/s12885-023-10929-9. 37259027 PMC10230804

[79] N. A. Blessing , S. Kasturirangan , E. M. Zink , A. L. Schroyer , and D. N. Chadee , “Osmotic and Heat Stress‐Dependent Regulation of MLK4β and MLK3 by the CHIP E3 Ligase in Ovarian Cancer Cells,” Cellular Signalling 39 (2017): 66–73, 10.1016/j.cellsig.2017.07.021. 28757353 PMC5592140

[80] D. Kramer , N. Stark , R. Schulz‐Heddergott , et al., “Strong Antitumor Synergy Between DNA Crosslinking and HSP90 Inhibition Causes Massive Premitotic DNA Fragmentation in Ovarian Cancer Cells,” Cell Death & Differentiation 24, no. 2 (2017): 300–316, 10.1038/cdd.2016.124. 27834954 PMC5299713

[81] R. Gabbasov , I. D. Benrubi , S. W. O'Brien , et al., “Targeted Blockade of HSP90 Impairs DNA‐damage Response Proteins and Increases the Sensitivity of Ovarian Carcinoma Cells to PARP Inhibition,” Cancer Biology & Therapy 20, no. 7 (2019): 1035–1045, 10.1080/15384047.2019.1595279. 30929564 PMC6606007

[82] A. Citarella , S. Belluti , D. Bonanni , et al., “Structure‐Based Discovery of Hsp90/HDAC6 Dual Inhibitors Targeting Aggressive Prostate Cancer,” Journal of Medicinal Chemistry 68, no. 15 (2025): 15738–15765, 10.1021/acs.jmedchem.5c00717. 40699153 PMC12362625

[83] A. Varga , M. T. Nguyen , K. Pénzes , et al., “Protein Kinase D3 (PKD3) Requires Hsp90 for Stability and Promotion of Prostate Cancer Cell Migration,” Cells 12, no. 2 (2023): 212, 10.3390/cells12020212. 36672148 PMC9857065

[84] K. D. Nolan , J. Kaur , and J. S. Isaacs , “Secreted Heat Shock Protein 90 Promotes Prostate Cancer Stem Cell Heterogeneity,” Oncotarget 8, no. 12 (2017): 19323–19341, 10.18632/oncotarget.14252. 28038472 PMC5386687

[85] T. Nakashima , T. Ishii , H. Tagaya , et al., “New Molecular and Biological Mechanism of Antitumor Activities of KW‐2478, a Novel Nonansamycin Heat Shock Protein 90 Inhibitor, in Multiple Myeloma Cells,” Clinical Cancer Research 16, no. 10 (2010): 2792–2802, 10.1158/1078-0432.CCR-09-3112. 20406843

[86] S. Ganguly , T. Home , A. Yacoub , et al., “Targeting HSF1 Disrupts HSP90 Chaperone Function in Chronic Lymphocytic Leukemia,” Oncotarget 6, no. 31 (2015): 31767–31779, 10.18632/oncotarget.5167. 26397138 PMC4741638

[87] L. Jia , H. Yang , Y. Liu , et al., “Targeted Delivery of HSP90 Inhibitors for Efficient Therapy of CD44‐Positive Acute Myeloid Leukemia and Solid Tumor‐Colon Cancer,” Journal of Nanobiotechnology 22, no. 1 (2024): 198, 10.1186/s12951-024-02460-1. 38649957 PMC11036589

[88] D. Zeng , M. Gao , R. Zheng , et al., “The HSP90 Inhibitor KW‐2478 Depletes the Malignancy of BCR/ABL and Overcomes the Imatinib‐Resistance Caused by BCR/ABL Amplification,” Experimental Hematology & Oncology 11, no. 1 (2022): 33, 10.1186/s40164-022-00287-w. 35624462 PMC9137153

[89] A. Bohush , P. Bieganowski , and A. Filipek , “Hsp90 and Its Co‐Chaperones in Neurodegenerative Diseases,” International Journal of Molecular Sciences 20, no. 20 (2019): 4976, 10.3390/ijms20204976. 31600883 PMC6834326

[90] H. Wu and H. J. Dyson , “Aggregation of Zinc‐Free p53 is Inhibited by Hsp90 But Not Other Chaperones,” Protein Science 28, no. 11 (2019): 2020–2023, 10.1002/pro.3726. 31503385 PMC6798128

[91] Y. Chen , B. Wang , D. Liu , et al., “Hsp90 Chaperone Inhibitor 17‐AAG Attenuates Aβ‐Induced Synaptic Toxicity and Memory Impairment,” The Journal of Neuroscience 34, no. 7 (2014): 2464–2470, 10.1523/JNEUROSCI.0151-13.2014. 24523537 PMC3921421

[92] N. L. Nguyen , T. X. Hoang , and J. Y. Kim , “All‐Trans Retinoic Acid‐Induced Cell Surface Heat Shock Protein 90 Mediates Tau Protein Internalization and Degradation in Human Microglia,” Molecular Neurobiology 62, no. 1 (2025): 742–755, 10.1007/s12035-024-04295-1. 38900367 PMC11711573

[93] W.‐J. Jang , S.‐K. Jung , J.‐S. Kang , et al., “Anti‐Tumor Activity of WK 88‐1, a Novel Geldanamycin Derivative, in Gefitinib‐Resistant Non‐Small Cell Lung Cancers With Met Amplification,” Cancer Science 105, no. 10 (2014): 1245–1253, 10.1111/cas.12497. 25117641 PMC4462346

[94] S. M. Roe , C. Prodromou , R. O'Brien , J. E. Ladbury , P. W. Piper , and L. H. Pearl , “Structural Basis for Inhibition of the Hsp90 Molecular Chaperone by the Antitumor Antibiotics Radicicol and Geldanamycin,” Journal of Medicinal Chemistry 42, no. 2 (1999): 260–266, 10.1021/jm980403y. 9925731

[95] M. Gorska , U. Popowska , A. Sielicka‐Dudzin , et al., “Geldanamycin and Its Derivatives as Hsp90 Inhibitors,” FBL 17, no. 6 (2012): 2269–2277. 22652777 10.2741/4050

[96] M. Waza , H. Adachi , M. Katsuno , M. Minamiyama , F. Tanaka , and G. Sobue , “Alleviating Neurodegeneration by an Anticancer Agent,” Annals of the New York Academy of Sciences 1086 (2006): 21–34, 10.1196/annals.1377.012. 17185503

[97] S. M. Raja , R. J. Clubb , M. Bhattacharyya , et al., “A Combination of Trastuzumab and 17‐AAG Induces Enhanced Ubiquitinylation and Lysosomal Pathway‐Dependent ErbB2 Degradation and Cytotoxicity in ErbB2‐Overexpressing Breast Cancer Cells,” Cancer Biology & Therapy 7, no. 10 (2008): 1630–1640, 10.4161/cbt.7.10.6585. 18769124 PMC2727620

[98] E. Hertlein , A. J. Wagner , J. Jones , et al., “17‐DMAG Targets the Nuclear Factor‐κB Family of Proteins to Induce Apoptosis in Chronic Lymphocytic Leukemia: Clinical Implications of HSP90 Inhibition,” Blood 116, no. 1 (2010): 45–53, 10.1182/blood-2010-01-263756. 20351313 PMC2904580

[99] C. Wong and S. Chen , “Heat Shock Protein 90 Inhibitors: New Mode of Therapy to Overcome Endocrine Resistance,” Cancer Research 69, no. 22 (2009): 8670–8677, 10.1158/0008-5472.CAN-09-1259. 19861537 PMC2784253

[100] C. Zhou , T. Yu , R. Zhu , et al., “Timosaponin AIII Promotes Non‐Small‐Cell Lung Cancer Ferroptosis Through Targeting and Facilitating HSP90 Mediated GPX4 Ubiquitination and Degradation,” International Journal of Biological Sciences 19, no. 5 (2023): 1471–1489, 10.7150/ijbs.77979. 37056925 PMC10086754

[101] Z. Wu , H. Zhuang , Q. Yu , et al., “Homoharringtonine Combined With the Heat Shock Protein 90 Inhibitor IPI504 in the Treatment of FLT3‐ITD Acute Myeloid Leukemia,” Translational Oncology 12, no. 6 (2019): 801–809, 10.1016/j.tranon.2019.02.016. 30953928 PMC6449739

[102] Q. Huang , S. He , Y. Tian , et al., “Hsp90 Inhibition Destabilizes Ezh2 Protein in Alloreactive T Cells and Reduces Graft‐Versus‐Host Disease in Mice,” Blood 129, no. 20 (2017): 2737–2748, 10.1182/blood-2016-08-735886. 28246193 PMC5437825

[103] M. Mohammadian , S. Feizollahzadeh , R. Mahmoudi , A. T. Milani , S. R. Firouzi , and B. K. Douna , “Hsp90 Inhibitor; NVP‐AUY922 in Combination With Doxorubicin Induces Apoptosis and Downregulates VEGF in MCF‐7 Breast Cancer Cell Line,” Asian Pac J Cancer Prev 21, no. 6 (2020): 1773–1778. 32592377 10.31557/APJCP.2020.21.6.1773 PMC7568890

[104] H. Taniguchi , H. Hasegawa , D. Sasaki , et al., “Heat Shock Protein 90 Inhibitor NVP ‐ AUY 922 Exerts Potent Activity Against Adult T‐Cell Leukemia–Lymphoma Cells,” Cancer Science 105, no. 12 (2014): 1601–1608, 10.1111/cas.12540. 25263741 PMC4317953

[105] W. C. Cho and C. F. Wong , “Potential Benefits of Combined Treatment With Hsp90 Inhibitor AUY922 and Cisplatin for Overcoming Drug Resistance in Nasopharyngeal Carcinoma,” American Journal of Cancer Research 15, no. 2 (2025): 533–545, 10.62347/OSGO7209. 40084353 PMC11897633

[106] M. L. Johnson , H. A. Yu , E. M. Hart , et al., “Phase I/II Study of HSP90 Inhibitor AUY922 and Erlotinib for EGFR‐Mutant Lung Cancer with Acquired Resistance to Epidermal Growth Factor Receptor Tyrosine Kinase Inhibitors,” Journal of Clinical Oncology 33, no. 15 (2015): 1666–1673, 10.1200/JCO.2014.59.7328. 25870087 PMC4881377

[107] Y. Wang , H. Liu , L. Diao , et al., “Hsp90 Inhibitor Ganetespib Sensitizes Non–Small Cell Lung Cancer to Radiation but Has Variable Effects With Chemoradiation,” Clinical Cancer Research 22, no. 23 (2016): 5876–5886, 10.1158/1078-0432.CCR-15-2190. 27354472 PMC5135582

[108] F. Chen , C. Tang , F. Yang , et al., “HSP90 inhibition Suppresses Tumor Glycolytic Flux to Potentiate the Therapeutic Efficacy of Radiotherapy for Head and Neck Cancer,” Science Advances 10, no. 8 (2024): adk3663, 10.1126/sciadv.adk3663. PMC10889358 38394204

[109] D. B. Cardin , R. Thota , L. W. Goff , et al., “A Phase II Study of Ganetespib as Second‐Line or Third‐Line Therapy for Metastatic Pancreatic Cancer,” American Journal of Clinical Oncology 41, no. 8 (2018): 772–776, 10.1097/COC.0000000000000377. 28301350 PMC5599313

[110] G. Chiosis , M. N. Timaul , B. Lucas , et al., “A Small Molecule Designed to Bind to the Adenine Nucleotide Pocket of Hsp90 Causes Her2 Degradation and the Growth Arrest and Differentiation of Breast Cancer Cells,” Chemistry & Biology 8, no. 3 (2001): 289–299, 10.1016/S1074-5521(01)00015-1. 11306353

[111] E. Caldas‐Lopes , L. Cerchietti , J. H. Ahn , et al., “Hsp90 Inhibitor PU‐H71, a Multimodal Inhibitor of Malignancy, Induces Complete Responses in Triple‐Negative Breast Cancer Models,” Proceedings of the National Academy of Sciences 106, no. 20 (2009): 8368–8373, 10.1073/pnas.0903392106. PMC2688867 19416831

[112] A. Guo , P. Lu , J. Lee , C. Zhen , G. Chiosis , and Y. L. Wang , “HSP90 Stabilizes B‐Cell Receptor Kinases in a Multi‐Client Interactome: PU‐H71 Induces CLL Apoptosis in a Cytoprotective Microenvironment,” Oncogene 36, no. 24 (2017): 3441–3449, 10.1038/onc.2016.494. 28114285 PMC5645670

[113] K. L. Jhaveri , C. H. Dos Anjos , T. Taldone , et al., “Measuring Tumor Epichaperome Expression Using [(124)I] PU‐H71 Positron Emission Tomography as a Biomarker of Response for PU‐H71 Plus Nab‐Paclitaxel in HER2‐Negative Metastatic Breast Cancer,” JCO Precis Oncol 4 (2020): PO2000273, 10.1200/PO.20.00273. PMC7713524 33283132

[114] H. A. Rothan , Y. Zhong , M. A. Sanborn , et al., “Small Molecule grp94 Inhibitors Block Dengue and Zika Virus Replication,” Antiviral Research 171 (2019): 104590, 10.1016/j.antiviral.2019.104590. 31421166 PMC6801034

[115] P. D. Patel , P. Yan , P. M. Seidler , et al., “Paralog‐Selective Hsp90 Inhibitors Define Tumor‐Specific Regulation of HER2,” Nature Chemical Biology 9, no. 11 (2013): 677–684, 10.1038/nchembio.1335. 23995768 PMC3982621

[116] P. Yan , H. J. Patel , S. Sharma , et al., “Molecular Stressors Engender Protein Connectivity Dysfunction Through Aberrant N‐Glycosylation of a Chaperone,” Cell Reports 31, no. 13 (2020): 107840, 10.1016/j.celrep.2020.107840. 32610141 PMC7372946

[117] X. Huang , W. Zhang , N. Yang , et al., “Identification of HSP90B1 in Pan‐Cancer Hallmarks to Aid Development of a Potential Therapeutic Target,” Molecular Cancer 23, no. 1 (2024): 19, 10.1186/s12943-023-01920-w. 38243263 PMC10799368

[118] R. Gopalakrishnan , H. Matta , and P. M. Chaudhary , “A Purine Scaffold HSP90 Inhibitor BIIB021 Has Selective Activity Against KSHV‐associated Primary Effusion Lymphoma and Blocks vFLIP K13‐induced NF‐κB,” Clinical Cancer Research 19, no. 18 (2013): 5016–5026, 10.1158/1078-0432.CCR-12-3510. 23881928 PMC3804723

[119] A. C. Lai and C. M. Crews , “Induced Protein Degradation: An Emerging Drug Discovery Paradigm,” Nature Reviews Drug Discovery 16, no. 2 (2017): 101–114, 10.1038/nrd.2016.211. 27885283 PMC5684876

[120] K. Pugh , H. Xu , and B. S. J. Blagg , “Investigation of the Site 2 Pocket of Grp94 With KUNG65 Benzamide Derivatives,” Bioorganic & Medicinal Chemistry Letters 111 (2024): 129893, 10.1016/j.bmcl.2024.129893. 39043265 PMC11385017

[121] A. Rajan , R. J. Kelly , J. B. Trepel , et al., “A Phase I Study of PF‐04929113 (SNX‐5422), an Orally Bioavailable Heat Shock Protein 90 Inhibitor, in Patients With Refractory Solid Tumor Malignancies and Lymphomas,” Clinical Cancer Research 17, no. 21 (2011): 6831–6839, 10.1158/1078-0432.CCR-11-0821. 21908572 PMC3207004

[122] H.‐H. Kim , J.‐S. Hyun , J. Choi , K.‐E. Choi , J.‐G. Jee , and S. J. Park , “Structural Ensemble‐Based Docking Simulation and Biophysical Studies Discovered New Inhibitors of Hsp90 N‐Terminal Domain,” Scientific Reports 8, no. 1 (2018): 368, 10.1038/s41598-017-18332-8. 29321504 PMC5762686

[123] J. A. Friedman , S. C. Wise , M. Hu , et al., “HSP90 Inhibitor SNX5422/2112 Targets the Dysregulated Signal and Transcription Factor Network and Malignant Phenotype of Head and Neck Squamous Cell Carcinoma,” Translational Oncology 6, no. 4 (2013): 429–441, 10.1593/tlo.13292. 23908686 PMC3730018

[124] Y. Saito , T. Takahashi , Y. Obata , et al., “TAS‐116 Inhibits Oncogenic KIT Signalling on the Golgi in Both Imatinib‐Naïve and Imatinib‐Resistant Gastrointestinal Stromal Tumours,” British Journal of Cancer 122, no. 5 (2020): 658–667, 10.1038/s41416-019-0688-y. 31857719 PMC7054534

[125] J. Liang , D. Wang , Y. Zhao , et al., “Novel Hsp90‐Targeting PROTACs: Enhanced Synergy With Cisplatin in Combination Therapy of Cervical Cancer,” European Journal of Medicinal Chemistry 275 (2024): 116572, 10.1016/j.ejmech.2024.116572. 38861809

[126] T. Doi , Y. Kurokawa , A. Sawaki , et al., “Efficacy and Safety of TAS‐116, an Oral Inhibitor of Heat Shock Protein 90, in Patients With Metastatic or Unresectable Gastrointestinal Stromal Tumour Refractory to Imatinib, Sunitinib and Regorafenib: A Phase II, Single‐Arm Trial,” European Journal of Cancer 121 (2019): 29–39, 10.1016/j.ejca.2019.08.009. 31536852

[127] H. E. Haarberg , K. H. T. Paraiso , E. Wood , et al., “Inhibition of Wee1, AKT, and CDK4 Underlies the Efficacy of the HSP90 Inhibitor XL888 in an In Vivo Model of NRAS‐mutant Melanoma,” Molecular Cancer Therapeutics 12, no. 6 (2013): 901–912, 10.1158/1535-7163.MCT-12-1003. 23538902 PMC3683468

[128] M. Sgobba , G. Degliesposti , A. M. Ferrari , and G. Rastelli , “Structural Models and Binding Site Prediction of the C‐Terminal Domain of Human Hsp90: A New Target for Anticancer Drugs,” Chemical Biology & Drug Design 71, no. 5 (2008): 420–433, 10.1111/j.1747-0285.2008.00650.x. 18373550

[129] B.‐H. Zhu , “(‐)‐Epigallocatechin‐3‐Gallate Inhibits VEGF Expression Induced by IL‐6 бvia Stat3 in Gastric Cancer,” World Journal of Gastroenterology 17, no. 18 (2011): 2315–2325, 10.3748/wjg.v17.i18.2315. PMC3098399 21633597

[130] B. N. Singh , S. Shankar , and R. K. Srivastava , “Green Tea Catechin, Epigallocatechin‐3‐Gallate (EGCG): Mechanisms, Perspectives and Clinical Applications,” Biochemical Pharmacology 82, no. 12 (2011): 1807–1821, 10.1016/j.bcp.2011.07.093. 21827739 PMC4082721

[131] N. M. Saeed , R. N. El‐Naga , W. M. El‐Bakly , H. M. Abdel‐Rahman , R. A. Salah ElDin , and E. El‐Demerdash , “Epigallocatechin‐3‐Gallate Pretreatment Attenuates Doxorubicin‐Induced Cardiotoxicity in Rats: A Mechanistic Study,” Biochemical Pharmacology 95, no. 3 (2015): 145–155, 10.1016/j.bcp.2015.02.006. 25701654

[132] A. Khandelwal , V. M. Crowley , and B. S. J. Blagg , “Natural Product Inspired N‐Terminal Hsp90 Inhibitors: From Bench to Bedside?,” Medicinal Research Reviews 36, no. 1 (2016): 92–118, 10.1002/med.21351. 26010985 PMC4659773

[133] G. Yang , T. Song , H. Zhang , et al., “Stimulus‐Detonated Biomimetic “Nanobomb” With Controlled Release of HSP90 Inhibitor to Disrupt Mitochondrial Function for Synergistic Gas and Photothermal Therapy,” Advanced Healthcare Materials 12, no. 26 (2023): 2300945, 10.1002/adhm.202300945. 37200205

[134] H. Zhao , G. E. Brandt , L. Galam , R. L. Matts , and B. S. J. Blagg , “Identification and Initial SAR of Silybin: An Hsp90 Inhibitor,” Bioorganic & Medicinal Chemistry Letters 21, no. 9 (2011): 2659–2664, 10.1016/j.bmcl.2010.12.088. 21273068 PMC12951813

[135] M. Taipale , I. Krykbaeva , M. Koeva , et al., “Quantitative Analysis of HSP90‐Client Interactions Reveals Principles of Substrate Recognition,” Cell 150, no. 5 (2012): 987–1001, 10.1016/j.cell.2012.06.047. 22939624 PMC3894786

[136] S. M. Roe , M. M. U. Ali , P. Meyer , et al., “The Mechanism of Hsp90 Regulation by the Protein Kinase‐Specific Cochaperone p50cdc37,” Cell 116, no. 1 (2004): 87–98, 10.1016/S0092-8674(03)01027-4. 14718169

[137] H. Hieronymus , J. Lamb , K. N. Ross , et al., “Gene Expression Signature‐Based Chemical Genomic Prediction Identifies a Novel Class of HSP90 Pathway Modulators,” Cancer Cell 10, no. 4 (2006): 321–330, 10.1016/j.ccr.2006.09.005. 17010675

[138] T. Zhang , Y. Li , Y. Yu , P. Zou , Y. Jiang , and D. Sun , “Characterization of Celastrol to Inhibit hsp90 and cdc37 Interaction,” Journal of Biological Chemistry 284, no. 51 (2009): 35381–35389, 10.1074/jbc.M109.051532. 19858214 PMC2790967

[139] S.‐R. Chen , Y. Dai , J. Zhao , L. Lin , Y. Wang , and Y. Wang , “A Mechanistic Overview of Triptolide and Celastrol, Natural Products From Tripterygium wilfordii Hook F,” Frontiers in Pharmacology 9 (2018): 104, 10.3389/fphar.2018.00104. 29491837 PMC5817256

[140] Y. Yu , A. Hamza , T. Zhang , et al., “Withaferin A Targets Heat Shock Protein 90 in Pancreatic Cancer Cells,” Biochemical Pharmacology 79, no. 4 (2010): 542–551, 10.1016/j.bcp.2009.09.017. 19769945 PMC2794909

[141] A. Grover , A. Shandilya , V. Agrawal , et al., “Hsp90/Cdc37 Chaperone/Co‐Chaperone Complex, a Novel Junction Anticancer Target Elucidated by the Mode of Action of Herbal Drug Withaferin A,” BMC Bioinformatics [Electronic Resource] 12, no. 1 (2011): S30. 21342561 10.1186/1471-2105-12-S1-S30 PMC3044286

[142] M. M. U. Ali , S. M. Roe , C. K. Vaughan , et al., “Crystal Structure of an Hsp90–Nucleotide–p23/Sba1 Closed Chaperone Complex,” Nature 440, no. 7087 (2006): 1013–1017, 10.1038/nature04716. 16625188 PMC5703407

[143] G. E. L. Brandt , M. D. Schmidt , T. E. Prisinzano , and B. S. J. Blagg , “Gedunin, a Novel Hsp90 Inhibitor: Semisynthesis of Derivatives and Preliminary Structure−Activity Relationships,” Journal of Medicinal Chemistry 51, no. 20 (2008): 6495–6502, 10.1021/jm8007486. 18816111 PMC2850591

[144] C. A. Patwardhan , A. Fauq , L. B. Peterson , C. Miller , B. S. J. Blagg , and A. Chadli , “Gedunin Inactivates the Co‐Chaperone p23 Protein Causing Cancer Cell Death by Apoptosis,” Journal of Biological Chemistry 288, no. 10 (2013): 7313–7325, 10.1074/jbc.M112.427328. 23355466 PMC3591639

[145] E. Ciglia , J. Vergin , S. Reimann , et al., “Resolving Hot Spots in the C‐Terminal Dimerization Domain That Determine the Stability of the Molecular Chaperone Hsp90,” PLoS ONE 9, no. 4 (2014): 96031, 10.1371/journal.pone.0096031. PMC3997499 24760083

[146] C. L. Carroll , J. V. C. Johnston , A. Kekec , et al., “Synthesis and Cytotoxicity of Novel Sansalvamide A Derivatives,” Organic Letters 7, no. 16 (2005): 3481–3484, 10.1021/ol051161g. 16048322

[147] R. P. Sellers , L. D. Alexander , V. A. Johnson , et al., “Design and Synthesis of Hsp90 Inhibitors: Exploring the SAR of Sansalvamide A Derivatives,” Bioorganic & Medicinal Chemistry 18, no. 18 (2010): 6822–6856, 10.1016/j.bmc.2010.07.042. 20708938 PMC2933939

[148] L. D. Alexander , J. R. Partridge , D. A. Agard , and S. R. McAlpine , “A Small Molecule That Preferentially Binds the Closed Conformation of Hsp90,” Bioorganic & Medicinal Chemistry Letters 21, no. 23 (2011): 7068–7071, 10.1016/j.bmcl.2011.09.096. 22014826

[149] R. C. Vasko , R. A. Rodriguez , C. N. Cunningham , V. C. Ardi , D. A. Agard , and S. R. McAlpine , “Mechanistic Studies of Sansalvamide A‐Amide: An Allosteric Modulator of Hsp90,” ACS Medicinal Chemistry Letters 1, no. 1 (2010): 4–8, 10.1021/ml900003t. 20730035 PMC2922868

[150] M. Tufail , C. H. Jiang , and N. Li , “Tumor Dormancy and Relapse: Understanding the Molecular Mechanisms of Cancer Recurrence,” Mil Med Res 12, no. 1 (2025): 7. 39934876 10.1186/s40779-025-00595-2 PMC11812268

[151] M. Vogt , N. Dienstbier , J. Schliehe‐Diecks , et al., “Co‐Targeting HSP90 Alpha and CDK7 Overcomes Resistance Against HSP90 Inhibitors in BCR‐ABL1+ Leukemia Cells,” Cell Death & Disease 14, no. 12 (2023): 799, 10.1038/s41419-023-06337-3. 38057328 PMC10700369

[152] X. Xiao , W. Wang , Y. Li , et al., “HSP90AA1‐Mediated Autophagy Promotes Drug Resistance in Osteosarcoma,” Journal of Experimental & Clinical Cancer Research 37, no. 1 (2018): 201, 10.1186/s13046-018-0880-6. 30153855 PMC6114771

[153] R. D. Cai , M. J. Lin , and Q. M. Ye , “Overcoming Cancer Drug Resistance Through Small‐Molecule Targeting of HSP90 and HSP70,” Cancer Drug Resistance 8 (2025): 60. 41425251 10.20517/cdr.2025.149 PMC12713180

[154] G. R. Bhat , I. Sethi , H. Q. Sadida , et al., “Cancer Cell Plasticity: From Cellular, Molecular, and Genetic Mechanisms to Tumor Heterogeneity and Drug Resistance,” Cancer and Metastasis Reviews 43, no. 1 (2024): 197–228, 10.1007/s10555-024-10172-z. 38329598 PMC11016008

[155] H.‐B. Kim , S.‐H. Lee , J.‐H. Um , et al., “Sensitization of Chemo‐Resistant Human Chronic Myeloid Leukemia Stem‐Like Cells to Hsp90 Inhibitor by SIRT1 Inhibition,” International Journal of Biological Sciences 11, no. 8 (2015): 923–934, 10.7150/ijbs.10896. 26157347 PMC4495410

[156] H. T. Le , H. T. Nguyen , H.‐Y. Min , et al., “Panaxynol, a Natural Hsp90 Inhibitor, Effectively Targets both Lung Cancer Stem and Non‐Stem Cells,” Cancer Letters 412 (2018): 297–307, 10.1016/j.canlet.2017.10.013. 29061506

[157] P. Singh and D. G. Jay , “The Role of eHsp90 in Extracellular Matrix Remodeling, Tumor Invasiveness, and Metastasis,” Cancers (Basel) 16, no. 22 (2024): 3873. 39594828 10.3390/cancers16223873 PMC11592750

[158] E. Cha , S. H. Hong , V. La , et al., “Ischemia‐induced Expression Status of Cofilin 1, CRSP2, HSP90, HSP27, and IL8 in Epicardial Adipose Tissue and Single Cell Transcriptomic Profiling of Stromal Cells,” Biochemistry and Cell Biology 103 (2025): 1–15, 10.1139/bcb-2024-0210. 39689294

[159] H. J. Lee , H.‐Y. Min , Y.‐S. Yong , et al., “A Novel C‐Terminal Heat Shock Protein 90 Inhibitor That Overcomes STAT3‐Wnt‐β‐Catenin Signaling‐Mediated Drug Resistance and Adverse Effects,” Theranostics 12, no. 1 (2022): 105–125, 10.7150/thno.63788. 34987637 PMC8690924

[160] G. Chakraborty , K. Gupta , and N. Kyprianou , “Epigenetic Mechanisms Underlying Subtype Heterogeneity and Tumor Recurrence in Prostate Cancer,” Nature Communications 14, no. 1 (2023): 567, 10.1038/s41467-023-36253-1. PMC9895058 36732329

[161] E. Lindell , L. Zhong , and X. Zhang , “Quiescent Cancer Cells—A Potential Therapeutic Target to Overcome Tumor Resistance and Relapse,” International Journal of Molecular Sciences 24, no. 4 (2023): 3762, 10.3390/ijms24043762. 36835173 PMC9959385

[162] C.‐S. Fan , C.‐C. Chen , L.‐L. Chen , et al., “Extracellular HSP90α Induces MyD88‐IRAK Complex‐Associated IKKα/β−NF‐κB/IRF3 and JAK2/TYK2−STAT‐3 Signaling in Macrophages for Tumor‐Promoting M2‐Polarization,” Cells 11, no. 2 (2022): 229, 10.3390/cells11020229. 35053345 PMC8774043

[163] V. Cabaud‐Gibouin , M. Durand , R. Quéré , F. Girodon , C. Garrido , and G. Jego , “Heat‐Shock Proteins in Leukemia and Lymphoma: Multitargets for Innovative Therapeutic Approaches,” Cancers 15, no. 3 (2023): 984, 10.3390/cancers15030984. 36765939 PMC9913431

[164] A. N. Kazakova , M. M. Lukina , K. S. Anufrieva , et al., “Exploring the Diversity of Cancer‐Associated Fibroblasts: Insights Into Mechanisms of Drug Resistance,” Frontiers in Cell and Developmental Biology 12 (2024): 1403122, 10.3389/fcell.2024.1403122. 38818409 PMC11137237

[165] S. Zhang , L. Yuan , L. Danilova , et al., “Spatial Transcriptomics Analysis of Neoadjuvant Cabozantinib and Nivolumab in Advanced Hepatocellular Carcinoma Identifies Independent Mechanisms of Resistance and Recurrence,” Genome Medicine 15, no. 1 (2023): 72, 10.1186/s13073-023-01218-y. 37723590 PMC10506285

[166] M. Z. Saleem , R. Huang , Y. Huang , et al., “Targeting TRAP1‐Dependent Metabolic Reprogramming to Overcome Doxorubicin Resistance in Quiescent Breast Cancer,” Drug Resistance Updates 81 (2025): 101226, 10.1016/j.drup.2025.101226. 40086176

[167] S. Chatterjee , E. H.‐B. Huang , I. Christie , B. F. Kurland , and T. F. Burns , “Acquired Resistance to the Hsp90 Inhibitor, Ganetespib, in KRAS‐ Mutant NSCLC Is Mediated via Reactivation of the ERK–p90RSK–mTOR Signaling Network,” Molecular Cancer Therapeutics 16, no. 5 (2017): 793–804, 10.1158/1535-7163.MCT-16-0677. 28167505 PMC5418121

[168] Q. Liu , G. Tu , Y. Hu , et al., “Discovery of BP3 as an Efficacious Proteolysis Targeting Chimera (PROTAC) Degrader of HSP90 for Treating Breast Cancer,” European Journal of Medicinal Chemistry 228 (2022): 114013, 10.1016/j.ejmech.2021.114013. 34864330

[169] Q. Wang , P. Liu , Y. Wen , et al., “Metal‐Enriched HSP90 Nanoinhibitor Overcomes Heat Resistance in Hyperthermic Intraperitoneal Chemotherapy Used for Peritoneal Metastases,” Molecular Cancer 22, no. 1 (2023): 95, 10.1186/s12943-023-01790-2. 37316830 PMC10265871

[170] Y. Yuan , Y. Tian , X. He , J. Dong , Z. Liu , and H. Jing , “HSP90 Inhibitor‐Loaded Hollow Mesoporous Nanoparticles for Enhanced Synergistic Mild Photothermal/Chemotherapy in Triple Negative Breast Cancer,” International Journal of Biological Macromolecules 310, no. Pt 1 (2025): 143266, 10.1016/j.ijbiomac.2025.143266. 40250687

[171] Y. Jin , Y. Huang , H. Ren , et al., “Nano‐Enhanced Immunotherapy: Targeting the Immunosuppressive Tumor Microenvironment,” Biomaterials 305 (2024): 122463, 10.1016/j.biomaterials.2023.122463. 38232643

[172] D. M. Quiroga , H. Savardekar , E. Schwarz , et al., “Abstract 7632: Final Survival Outcomes and Post‐hoc Tumor Gene Expression Pathway Analyses of Complete Responders From a Phase Ib Clinical Trial of HSP90 Inhibitor onalespib and Paclitaxel in Patients With Advanced Triple‐Negative Breast Cancer,” Cancer Research 84, no. 6 (2024): 7632–7632, 10.1158/1538-7445.AM2024-7632.

[173] N. O. Williams , D. Quiroga , C. Johnson , et al., “Phase Ib Study of HSP90 Inhibitor, Onalespib (AT13387), in Combination With Paclitaxel in Patients With Advanced Triple‐Negative Breast Cancer,” Therapeutic Advances in Medical Oncology 15 (2023): 17588359231217976, 10.1177/17588359231217976. 38152697 PMC10752118

[174] Ö. Kaplan and N. Göksen Tosun , “Molecular Pathway of Anticancer Effect of Next‐Generation HSP90 Inhibitors XL‐888 and Debio0932 in Neuroblastoma Cell Line,” Medical Oncology 41, no. 8 (2024): 194, 10.1007/s12032-024-02428-z. 38958814 PMC11222184

[175] G. A. Tehrani , B. Kubick , A. Gutierrez , et al., “Abstract 85: Abstract 823: Combination Therapy of HDAC Inhibitors With HSP90 and TRAP1 Targeting the Hypoxia Pathway in Triple‐Negative Breast Cancer,” Cancer Research 85, no. 8 (2025): 823–823, 10.1158/1538-7445.AM2025-823.

[176] H. Muraoka , H. Kazuno , A. Hashimoto , H. Sootome , and S. Ohkubo , “Pimitespib, an HSP90 Inhibitor, Enhances the Efficacy of PARP Inhibitors in PARP Inhibitor‐Insensitive Breast Cancer Cells,” Cancer Science 116, no. 6 (2025): 1745–1757, 10.1111/cas.70058. 40167031 PMC12127109

[177] Y. Kawamoto , H. Nakajima , Y. Komatsu , et al., “A Phase 1 Study of PARP Inhibitor (niraparib) Plus HSP90 Inhibitor (pimitespib) in Solid Tumors: Dose‐Expansion Results From the NiraPim (EPOC2102) Study,” Journal of Clinical Oncology 43, no. 16 (2025): 3079–3079, 10.1200/JCO.2025.43.16_suppl.3079.

[178] W. S. El‐Deiry , S. L. Graff , C. G. Azzoli , et al., “BrUOG 387: Phase Ib Investigator‐Initiated Trial of a Heat Shock Protein 90 Inhibitor (HSP90i) Combined With a CDK4/6i in Advanced Breast Cancer Progressing on CDK4/6i and in Solid Tumors With Retinoblastoma (Rb)‐Deficiency (IND163592),” Journal of Clinical Oncology 41, no. 16 (2023): TPS3167–TPS3167.

[179] P. Suwannalert , P. Panpinyaporn , P. Wantanachaisaeng , et al., “A Potential Combination of Targeting HSP90 and mTOR in Breast Cancer Cell Growth, Migration, and Invasion through Inhibiting AKT Phosphorylation and F‐actin Organization,” Anticancer Research 44, no. 6 (2024): 2555–2565, 10.21873/anticanres.17061. 38821604

[180] S. Lee , J. Jung , Y.‐J. Lee , et al., “Targeting HSF1 as a Therapeutic Strategy for Multiple Mechanisms of EGFR Inhibitor Resistance in EGFR Mutant Non‐Small‐Cell Lung Cancer,” Cancers 13, no. 12 (2021): 2987, 10.3390/cancers13122987. 34203709 PMC8232331

[181] S. Li , Z. Chen , W. Guo , et al., “H_2_S‐Mediated Gas Therapy and HSP90 Downregulation Synergically Enhance Tumor Microwave Thermal Therapy,” Advanced Functional Materials 34, no. 21 (2024): 2314742, 10.1002/adfm.202314742.

[182] M. A. Bylicky , U. Shankavaram , M. J. Aryankalayil , et al., “Multiomic‐Based Molecular Landscape of FaDu Xenograft Tumors in Mice After a Combinatorial Treatment With Radiation and an HSP90 Inhibitor Identifies Adaptation‐Induced Targets of Resistance and Therapeutic Intervention,” Molecular Cancer Therapeutics 23, no. 4 (2024): 577–588, 10.1158/1535-7163.MCT-23-0796. 38359816 PMC10985469

[183] A. S. Lee , “Glucose‐Regulated Proteins in Cancer: Molecular Mechanisms and Therapeutic Potential,” Nature Reviews Cancer 14, no. 4 (2014): 263–276, 10.1038/nrc3701. 24658275 PMC4158750

[184] J. E. Lee , P. I. Cathey , H. Wu , R. Parker , and G. K. Voeltz , “Endoplasmic Reticulum Contact Sites Regulate the Dynamics of Membraneless Organelles,” Science 367, no. 6477 (2020): aay7108, 10.1126/science.aay7108. PMC10088059 32001628

[185] D. Ron and P. Walter , “Signal Integration in the Endoplasmic Reticulum Unfolded Protein Response,” Nature Reviews Molecular Cell Biology 8, no. 7 (2007): 519–529, 10.1038/nrm2199. 17565364

[186] Y. Yang , B. Liu , J. Dai , et al., “Heat Shock Protein gp96 Is a Master Chaperone for Toll‐Like Receptors and Is Important in the Innate Function of Macrophages,” Immunity 26, no. 2 (2007): 215–226, 10.1016/j.immuni.2006.12.005. 17275357 PMC2847270

[187] B. Liu , M. Staron , F. Hong , et al., “Essential Roles of grp94 in Gut Homeostasis via Chaperoning Canonical Wnt Pathway,” Proceedings of the National Academy of Sciences 110, no. 17 (2013): 6877–6882, 10.1073/pnas.1302933110. PMC3637754 23572575

[188] Y. Zhang , B. X. Wu , A. Metelli , et al., “GP96 is a GARP Chaperone and Controls Regulatory T Cell Functions,” Journal of Clinical Investigation 125, no. 2 (2015): 859–869, 10.1172/JCI79014. 25607841 PMC4319419

[189] E. A. Ansa‐Addo , J. Thaxton , F. Hong , et al., “Clients and Oncogenic Roles of Molecular Chaperone gp96/grp94,” Current Topics in Medicinal Chemistry 16, no. 25 (2016): 2765–2778, 10.2174/1568026616666160413141613. 27072698 PMC5041304

[190] R. A. Sager , F. Khan , L. Toneatto , et al., “Targeting Extracellular Hsp90: A Unique Frontier Against Cancer,” Frontiers in Molecular Biosciences 9 (2022): 982593, 10.3389/fmolb.2022.982593. 36060252 PMC9428293

[191] Y. Zhou , Z. Zhong , and P. Hu , “Chronic ER Stress Triggers Cell‐Surface Chaperones as the Therapeutic Targets of CAR Cells in Acute Myeloid Leukemia,” Advanced Science 13, no. 5 (2026): 11573. 10.1002/advs.202511573 PMC12849903 41126722

[192] T. Eguchi , C. Sogawa , K. Ono , et al., “Cell Stress Induced Stressome Release Including Damaged Membrane Vesicles and Extracellular HSP90 by Prostate Cancer Cells,” Cells 9, no. 3 (2020): 755. 32204513 10.3390/cells9030755 PMC7140686

[193] S. Munro and H. R. Pelham , “A C‐terminal Signal Prevents Secretion of Luminal ER Proteins,” Cell 48, no. 5 (1987): 899–907, 10.1016/0092-8674(87)90086-9. 3545499

[194] N. Dean and H. R. Pelham , “Recycling of Proteins From the Golgi Compartment to the ER in Yeast,” The Journal of cell biology 111, no. 2 (1990): 369–377, 10.1083/jcb.111.2.369. 2199456 PMC2116185

[195] H. Takemoto , T. Yoshimori , A. Yamamoto , et al., “Heavy Chain Binding Protein (BiP/GRP78) and Endoplasmin Are Exported From the Endoplasmic Reticulum in Rat Exocrine Pancreatic Cells, Similar to Protein Disulfide‐isomerase,” Archives of Biochemistry and Biophysics 296, no. 1 (1992): 129–136, 10.1016/0003-9861(92)90554-A. 1318687

[196] J. Melnick , S. Aviel , and Y. Argon , “The Endoplasmic Reticulum Stress Protein GRP94, in Addition to BiP, Associates With Unassembled Immunoglobulin Chains,” Journal of Biological Chemistry 267, no. 30 (1992): 21303–21306, 10.1016/S0021-9258(19)36608-6. 1400441

[197] J. Helenius and M. Aebi , “Transmembrane Movement of Dolichol Linked Carbohydrates During N‐glycoprotein Biosynthesis in the Endoplasmic Reticulum,” Seminars in Cell & Developmental Biology 13, no. 3 (2002): 171–178, 10.1016/S1084-9521(02)00045-9. 12137737

[198] M. Z. Ahmed and A. S. Alqahtani , “Cell Surface Expression of Ribophorin I, an Endoplasmic Reticulum Protein, Over Different Cell Types,” International Journal of Biological Macromolecules 264, no. Pt 1 (2024): 130278, 10.1016/j.ijbiomac.2024.130278. 38373565

[199] Y.‐L. Tsai , D. P. Ha , H. Zhao , et al., “Endoplasmic Reticulum Stress Activates SRC, Relocating Chaperones to the Cell Surface Where GRP78/CD109 Blocks TGF‐β Signaling,” Proceedings of the National Academy of Sciences 115, no. 18 (2018): E4245–E4254, 10.1073/pnas.1714866115. PMC5939063 29654145

[200] J. W. Kim , Y. B. Cho , and S. Lee , “Cell Surface GRP94 as a Novel Emerging Therapeutic Target for Monoclonal Antibody Cancer Therapy,” Cells 10, no. 3 (2021): 670, 10.3390/cells10030670. 33802964 PMC8002708

[201] H. J. Kim , Y. B. Cho , K. Heo , et al., “Targeting Cell Surface Glucose‐Regulated Protein 94 in Gastric Cancer With an Anti‐GRP94 Human Monoclonal Antibody,” BMB Reports 57, no. 4 (2024): 188–193, 10.5483/BMBRep.2023-0195. 38449302 PMC11058359

[202] S. Xia , W. Duan , W. Liu , X. Zhang , and Q. Wang , “GRP78 in Lung Cancer,” Journal of Translational Medicine 19, no. 1 (2021): 118, 10.1186/s12967-021-02786-6. 33743739 PMC7981903

[203] M. Wickenberg , R. Mercier , M. Yap , J. Walker , K. Baker , and P. LaPointe , “Hsp90 Inhibition Leads to an Increase in Surface Expression of Multiple Immunological Receptors in Cancer Cells,” Frontiers in Molecular Biosciences 11 (2024): 1334876, 10.3389/fmolb.2024.1334876. 38645275 PMC11027010

[204] T. Imai , Y. Kato , C. Kajiwara , et al., “Heat Shock Protein 90 (HSP90) Contributes to Cytosolic Translocation of Extracellular Antigen for Cross‐Presentation by Dendritic Cells,” Proceedings of the National Academy of Sciences 108, no. 39 (2011): 16363–16368, 10.1073/pnas.1108372108. PMC3182735 21930907

[205] J. Kunisawa and N. Shastri , “Hsp90α Chaperones Large C‐Terminally Extended Proteolytic Intermediates in the MHC Class I Antigen Processing Pathway,” Immunity 24, no. 5 (2006): 523–534, 10.1016/j.immuni.2006.03.015. 16713971

[206] T. Ichiyanagi , T. Imai , C. Kajiwara , et al., “Essential Role of Endogenous Heat Shock Protein 90 of Dendritic Cells in Antigen Cross‐Presentation,” The Journal of Immunology 185, no. 5 (2010): 2693–2700, 10.4049/jimmunol.1000821. 20668218

[207] M. K. Callahan , M. Garg , and P. K. Srivastava , “Heat‐Shock Protein 90 Associates With N‐Terminal Extended Peptides and Is Required for Direct and Indirect Antigen Presentation,” Proceedings of the National Academy of Sciences 105, no. 5 (2008): 1662–1667, 10.1073/pnas.0711365105. PMC2234201 18216248

[208] K. Liu , J. Huang , J. Liu , et al., “HSP90 Mediates IFNγ‐Induced Adaptive Resistance to Anti‐PD‐1 Immunotherapy,” Cancer Research 82, no. 10 (2022): 2003–2018, 10.1158/0008-5472.CAN-21-3917. 35247909

[209] Z. Zhang , D. Sun , H. Tang , J. Ren , S. Yin , and K. Yang , “PER2 Binding to HSP90 Enhances Immune Response Against Oral Squamous Cell Carcinoma by Inhibiting IKK/NF‐κB Pathway and PD‐L1 Expression,” Journal for ImmunoTherapy of Cancer 11, no. 11 (2023): 007627, 10.1136/jitc-2023-007627. PMC10626827 37914384

[210] S. Rahmy , S. J. Mishra , S. Murphy , B. S. J. Blagg , and X. Lu , “Hsp90β inhibition Upregulates Interferon Response and Enhances Immune Checkpoint Blockade Therapy in Murine Tumors,” Frontiers in Immunology 13 (2022): 1005045, 10.3389/fimmu.2022.1005045. 36341371 PMC9630337

[211] C. Tay , A. Tanaka , and S. Sakaguchi , “Tumor‐Infiltrating Regulatory T Cells as Targets of Cancer Immunotherapy,” Cancer Cell 41, no. 3 (2023): 450–465, 10.1016/j.ccell.2023.02.014. 36917950

[212] A. Kawazoe , K. Itahashi , N. Yamamoto , et al., “TAS‐116 (Pimitespib), an Oral HSP90 Inhibitor, in Combination With Nivolumab in Patients With Colorectal Cancer and Other Solid Tumors: An Open‐Label, Dose‐Finding, and Expansion Phase Ib Trial (EPOC1704),” Clinical Cancer Research 27, no. 24 (2021): 6709–6715, 10.1158/1078-0432.CCR-21-1929. 34593531

[213] A. Tsuge , S. Watanabe , A. Kawazoe , et al., “The HSP90 Inhibitor Pimitespib Targets Regulatory T Cells in the Tumor Microenvironment,” Cancer Immunology Research 13, no. 2 (2025): 273–285, 10.1158/2326-6066.CIR-24-0713. 39602577

[214] Z. Albakova , Y. Mangasarova , and A. Sapozhnikov , “Impaired Heat Shock Protein Expression in Activated T Cells in B‐Cell Lymphoma,” Biomedicines 10, no. 11 (2022): 2747, 10.3390/biomedicines10112747. 36359267 PMC9687880

[215] C. Mall , G. D. Sckisel , D. A. Proia , et al., “Repeated PD‐1/PD‐L1 Monoclonal Antibody Administration Induces Fatal Xenogeneic Hypersensitivity Reactions in a Murine Model of Breast Cancer,” Oncoimmunology 5, no. 2 (2016): 1075114, 10.1080/2162402X.2015.1075114. PMC4801432 27057446

[216] V. E. Mekers , V. M. Kho , M. Ansems , and G. J. Adema , “cGAS/cGAMP/STING Signal Propagation in the Tumor Microenvironment: Key Role for Myeloid Cells in Antitumor Immunity,” Radiotherapy and Oncology 174 (2022): 158–167, 10.1016/j.radonc.2022.07.014. 35870728

[217] J. Li , X. Han , M. Sun , et al., “Caspase‐9 Inhibition Triggers Hsp90‐Based Chemotherapy‐Mediated Tumor Intrinsic Innate Sensing and Enhances Antitumor Immunity,” Journal for ImmunoTherapy of Cancer 11, no. 12 (2023): 007625, 10.1136/jitc-2023-007625. PMC10711858 38056894

[218] G.‐L. He , Z. Luo , T.‐T. Shen , et al., “Inhibition of HSP90β by Ganetespib Blocks the Microglial Signalling of Evoked Pro‐Inflammatory Responses to Heat Shock,” The International Journal of Biochemistry & Cell Biology 106 (2019): 35–45, 10.1016/j.biocel.2018.11.003. 30448425

[219] J. Kim , M. Kwon , D. Park , et al., “HSP90 Inhibition Disrupts 27‐Hydroxycholesterol‐Induced Inflammatory Signaling in Monocytic Cells,” International Journal of Molecular Sciences 26, no. 20 (2025): 9963, 10.3390/ijms26209963. 41155259 PMC12563181

[220] B.‐Z. Qian , J. Li , H. Zhang , et al., “CCL2 Recruits Inflammatory Monocytes to Facilitate Breast‐Tumour Metastasis,” Nature 475, no. 7355 (2011): 222–225, 10.1038/nature10138. 21654748 PMC3208506

[221] S. L. Highfill , Y. Cui , A. J. Giles , et al., “Disruption of CXCR2‐Mediated MDSC Tumor Trafficking Enhances Anti‐PD1 Efficacy,” Science Translational Medicine 6, no. 237 (2014): 237ra267, 10.1126/scitranslmed.3007974. PMC6980372 24848257

[222] S. Saber , E. E. A. El‐Fattah , A. M. Abdelhamid , et al., “RETRACTED: Innovative Challenge for the Inhibition of Hepatocellular Carcinoma Progression by Combined Targeting of HSP90 and STAT3/HIF‐1α Signaling,” Biomedicine & Pharmacotherapy 158 (2023): 114196, 10.1016/j.biopha.2022.114196. 36916405

[223] N. Janssen , L. Speigl , G. Pawelec , H. Niessner , and C. Shipp , “Inhibiting HSP90 Prevents the Induction of Myeloid‐derived Suppressor Cells by Melanoma Cells,” Cellular Immunology 327 (2018): 68–76, 10.1016/j.cellimm.2018.02.012. 29478948

[224] M.‐S. Lee , S. M. Park , and Y.‐J. Kim , “Photothermal Treatment‐Based Heat Stress Regulates Function of Myeloid‐Derived Suppressor Cells,” Scientific Reports 14, no. 1 (2024): 18847, 10.1038/s41598-024-69074-3. 39143087 PMC11324874

[225] Y. Wang , A. Ma , N.‐J. Song , et al., “Proteotoxic Stress Response Drives T Cell Exhaustion and Immune Evasion,” Nature 647 (2025): 1025–1035, 10.1038/s41586-025-09539-1. 41034580 PMC12657239

[226] B. Zhang , M. Yang , W. Zhang , et al., “Chimeric Antigen Receptor‐Based Natural Killer Cell Immunotherapy in Cancer: From Bench to Bedside,” Cell Death & Disease 15, no. 1 (2024): 50, 10.1038/s41419-024-06438-7. 38221520 PMC10788349

[227] N. Hebbar , R. Epperly , A. Vaidya , et al., “CAR T Cells Redirected to Cell Surface GRP78 Display Robust Anti‐Acute Myeloid Leukemia Activity and Do Not Target Hematopoietic Progenitor Cells,” Nature Communications 13, no. 1 (2022): 587, 10.1038/s41467-022-28243-6. PMC8803836 35102167

[228] J. Ibanez , N. Hebbar , U. Thanekar , et al., “GRP78‐CAR T Cell Effector Function Against Solid and Brain Tumors is Controlled by GRP78 Expression on T cells,” Cell Reports Medicine 4, no. 11 (2023): 101297, 10.1016/j.xcrm.2023.101297. 37992682 PMC10694756

[229] S. Wang , W. Wei , Y. Yuan , J. Guo , D. Liang , and X. Zhao , “Cell‐Surface GRP78‐Targeted Chimeric Antigen Receptor T Cells Eliminate Lung Cancer Tumor Xenografts,” International Journal of Molecular Sciences 25, no. 1 (2024): 564, 10.3390/ijms25010564. 38203736 PMC10779323

[230] R. B. Zavareh , S. H. Spangenberg , A. Woods , F. Martínez‐Peña , and L. L. Lairson , “HSP90 Inhibition Enhances Cancer Immunotherapy by Modulating the Surface Expression of Multiple Immune Checkpoint Proteins,” Cell Chemical Biology 28, no. 2 (2021): 158–168, 10.1016/j.chembiol.2020.10.005. 33113406

[231] I. Skrabalak , A. Rajtak , B. Malachowska , et al., “Therapy Resistance: Modulating Evolutionarily Conserved Heat Shock Protein Machinery in Cancer,” Cancer Letters 616 (2025): 217571, 10.1016/j.canlet.2025.217571. 39986370

